# The SNL Histone Deacetylase‐Binding Factor GmHE13 Is a Novel Regulator of Soybean Hypocotyl Elongation

**DOI:** 10.1111/pbi.70310

**Published:** 2025-08-12

**Authors:** Zhikang Shen, Lili Zhuang, Wenqian Zhang, Feifei Liang, Yaqi Peng, Meng Yuan, Wei Zhang, Panpan Zhang, Xiangtao Li, Wei Wang, Min Chen

**Affiliations:** ^1^ State Key Laboratory of Crop Stress Adaptation and Improvement Henan University Zhengzhou China; ^2^ College of Life Science and Technology Huazhong Agricultural University Wuhan China; ^3^ Zhongzhou Laboratory of Integrative Biology Henan University Zhengzhou China; ^4^ Kaifeng Academy of Agriculture and Forestry Kaifeng China; ^5^ Biotechnology Developing Center Henan Academy of Sciences Zhengzhou China

**Keywords:** derepression, *expansin*, histone deacetylase complex, hypocotyl elongation, Sin3‐like protein, soybean

## Abstract

Hypocotyl elongation is one of the key events during seed germination and seedling establishment, determining the field performance and yield of soybean. In this study, using a genome‐wide association study (GWAS) method, we identified a candidate gene regulating soybean hypocotyl elongation, *GmHE13* (*HYPOCOTYL ELONGATION 13*), which encodes an SNL histone deacetylase (HDAC)‐binding protein. Knockout of *GmHE13* led to over‐elongated hypocotyls, while overexpression of *GmHE13* suppressed hypocotyl elongation, confirming GmHE13 as an inhibitor of hypocotyl development. As an SNL protein, GmHE13 interacts with multiple HDACs and a Myb‐like transcription factor (TF) GmRVE5 (REVEILLE5) to form HDAC complexes. These complexes remove H3K14 acetylation from hypocotyl growth‐related genes, including *expansins*, thereby repressing their expression and inhibiting hypocotyl elongation. Furthermore, the lead SNP (single‐nucleotide polymorphism) dividing *GmHE13* into two major haplotypes was found to determine the binding activity of a light‐responsive TF, GmOBP3, to *GmHE13's* promoter and lead to differential expression of *GmHE13*, resulting in differential hypocotyl elongation. Interestingly, the distribution of *GmHE13* haplotypes displayed a latitudinal preference, with *GmHE13* ^*Hap1*^ being a target of artificial selection during soybean breeding. Taken together, our findings revealed a novel epigenetic mechanism regulating soybean hypocotyl elongation, providing a theoretical basis and gene loci for directional improvement of soybean varieties.

## 1 Background

For dicots with epigeal germination such as soybean ( *Glycine max* [L.] Merr.), hypocotyls not only serve as physical support and facilitate nutrient transport, but also play a crucial role in pushing the cotyledons above the soil surface during seed germination, allowing the shoot to access light and initiate photosynthesis (Ahammed et al. 2020). Too short hypocotyls may fail the shoot to reach the light and survive. In this case, identifying quantitative trait loci (QTLs) and causal genes regulating soybean hypocotyl elongation, so as to develop varieties with proper and robust hypocotyl growth, is of great significance for improving seeding emergence and field performance. However, so far, studies on the molecular mechanisms underlying hypocotyl elongation were mainly carried out in model species such as *Arabidopsis thaliana* (hereafter *Arabidopsis*). The identified genes and regulatory routes are difficult to be directly transplanted into dicot crops such as soybean due to genome differences. The regulation mechanisms at the molecular level in crops including soybean thus remain largely elusive.

Hypocotyl elongation is regulated by interrelated factors, including environmental signals and internal molecular mechanisms, as shown by numerous studies in *Arabidopsis* (Al‐Sady et al. 2006; Yu et al. 2013). Besides, phytohormones such as gibberellins (GAs) play vital roles in the regulation of hypocotyl elongation (Li et al. 2016; Su et al. 2024). The stimulation of cell elongation by GAs and environmental signals is thought to be dependent on the expansion of the cell wall. However, the underlying mechanism is not very clear, which may involve cell‐wall loosening proteins and enzymes, including expansins and xyloglucan endotransglycosylases (XETs), as shown by studies in lettuce, cucumber, and tall fescue (McQueen‐Mason et al. 1992; Wagstaff et al. 2010; Xu et al. 2016). Expansins are a class of cell‐wall proteins that are widely present across species, allowing turgor‐driven expansion by influencing cell‐wall extensibility (Cosgrove 2015). Several studies suggested that *expansin* genes act downstream of light and phytohormones to regulate organ elongation. For example, in rice, *Os‐EXP4* is induced by GAs to be involved in the rapid internode elongation (Cho and Kende 1997). In soybean, the expression of *expansin* genes is regulated by light, cytokinin, and auxin, and is associated with hypocotyl elongation (Downes et al. 2001; Shen and Chen 2022).

In plants, acetylation of histone lysine residues is considered a mark of transcription activation, regulating various developmental processes (Tang et al. 2017; Wakeel et al. 2017). The balance of histone acetylation is mediated by histone acetyltransferases (HATs) and histone deacetylases (HDACs). HDACs remove acetyl groups from lysine residues of histones to inactivate gene expression. Studies have shown that HDACs interact with diverse chromatin and TFs to form histone deacetylase complexes such as the Sin3‐HDAC complex, playing a vital role in the epigenetic regulation of gene expression (Asmamaw et al. 2024; Jing et al. 2021). Sin3 (SWI‐independent3) is a highly conserved and multidomain‐containing scaffolding protein, providing a platform for the assembly of the core HDAC complex (Asmamaw et al. 2024). In *Arabidopsis*, there are six homologues of Sin3, named Sin3‐LIKEs (SNLs), which were reported to play key regulatory roles in multiple growth and development processes (Huang et al. 2019; Jing et al. 2021; Wang et al. 2013, 2016).

Hypocotyl elongation has significant effects on the field performance and yield of soybean. In this study, through GWAS, we identified a novel regulator of soybean hypocotyl elongation, GmHE13. We show that GmHE13 is a negative regulator of hypocotyl elongation. As a SNL family protein, GmHE13 recruits HDACs and a MYB TF, GmRVE5, to form an HDAC complex, thereby addressing and removing histone acetylation on *expansin* genes to inhibit their expression and subsequently suppress hypocotyl elongation. Our data clarified a novel epigenetic mechanism regulating soybean hypocotyl elongation, providing new insights into the role and regulatory mechanism of histone acetylation in legume growth and development.

## 2 Results

### 2.1 GWAS Identified the *qHE13* Locus Associated With Hypocotyl Elongation

Hypocotyl elongation plays a critical role in post‐germination development and field emergence of soybean seedlings. To clarify the genetic basis underlying hypocotyl elongation in soybean, we performed GWAS using a natural soybean population (Zhang et al. 2021). Hypocotyl lengths were measured for seeds harvested from independent experimental sites in Shijiazhuang, China, with three replicates. We observed a remarkable spectrum of variation in hypocotyl length within the population, ranging from 2.40 to 14.22 cm (Table S1), which was normally distributed and suitable for further association analysis (Figure S1).

**FIGURE 1:**
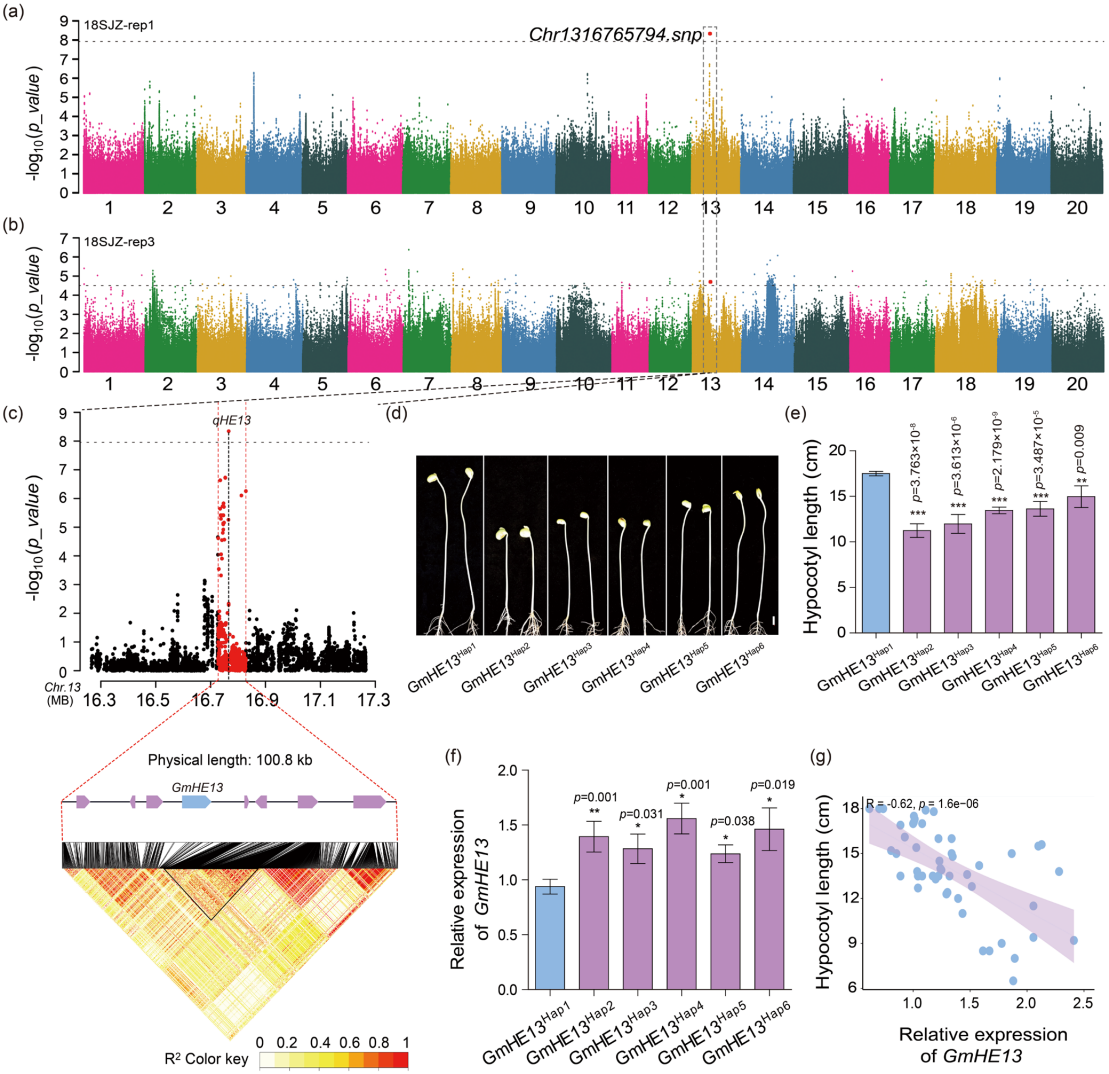
GWAS results indicate *qHE13* as a major locus associated with soybean hypocotyl length. (a, b) Manhattan plots depicting the GWAS results from replicate 1 (a) and replicate 3 (b) of 18SJZ soybean population. The red dots indicate the lead SNP, while the dotted lines indicate the significance threshold, with *p* = 1.21 × 10^−8^ in (a) and *p* = 2.42 × 10^−5^ in (b), respectively. (c) The local Manhattan plot of *qHE13*, spanning 100.8 kb region surrounding the lead SNP. The red dots indicate the SNPs within *qHE13*. The bottom diagram is the LD heatmap of SNPs with *qHE13*. The pairwise LD between the SNP markers is indicated as *R* ^2^ values, where red indicates a value of 1 and yellow indicates 0. (d) Representative photos of hypocotyls carrying different haplotypes of *GmHE13* grown under dark conditions. Scale bar, 1 cm. (e) Quantification of hypocotyl lengths in (d). Each haplotype is with nine randomly selected accessions. (f) RT‐qPCR results showing the relatively lower *GmHE13* expression in *GmHE13* ^*Hap1*^ hypocotyls grown under dark conditions. Each haplotype is with three randomly selected accessions. (g) Correlation between hypocotyl lengths and expression levels of *GmHE13*. Data represent mean ± SD. ***p < 0.001, **p < 0.01, *p < 0.05 (Student's *t*‐test).

GWAS was then performed with the mixed linear model (MLM). In the GWAS results, we noticed a lead SNP (*Chr1316765794.snp*) in chromosome 13 that could be found repeatedly (Figure 1a,b). Through the linkage disequilibrium (LD) block analysis, we defined an LD block region spanning 100.8 kb (*R* ^2^ > 0.6) (Table S2), which was named *qHE13* (*QTL HYPOCOTYL ELONGATION 13*). *qHE13* contains eight protein‐coding genes in the Wm82 v2.1 genome (Figure 1c). Among them, *Glyma.13G067800* and *Glyma.13G068000* showed robust expression in dry seeds based on our prior transcriptomic data, with their expression levels gradually decreasing during hypocotyl elongation. In contrast, other genes exhibited relatively low expression throughout hypocotyl elongation, making them less likely to be considered candidate genes (Figure S2a). For *Glyma.13G068000*, although haplotype analysis revealed that it segregates into two distinct haplotypes due to a missense mutation in its first exon, no significant difference in hypocotyl elongation was observed between them, suggesting that this gene is not associated with hypocotyl elongation (Figure S2b). Unlike *Glyma.13G068000*, *Glyma.13G067800* formed six haplotypes (Hap1—Hap6) based on 15 SNPs and 3 InDels (Figure S3a). Remarkably, soybean lines harbouring the *GmHE13* ^*Hap1*^ haplotype showed pronounced hypocotyl elongation under both light (Figure 1d,e) and dark conditions (Figure S3b,c) compared to those with alternative haplotypes, indicating that the distinctive SNPs in this haplotype are genetically linked to the hypocotyl elongation phenotype. Thus, *Glyma.13G067800* was designated as the candidate gene *GmHE13*.

### 2.2 GmHE13 Is a Negative Regulator of Hypocotyl Elongation

Notably, the lead SNP of *qHE13* is located in the 5ʹUTR of *GmHE13* (Figure S3a), distinguishing *GmHE13* ^*Hap1*^ from other haplotypes, which suggests that the lead SNP may underlie the observed hypocotyl length variation. Since promoter/5ʹUTR SNPs often affect gene expression, we analysed transcript levels and found that *GmHE13* ^*Hap1*^ accessions exhibited significantly reduced *GmHE13* expression in hypocotyls grown under both light and dark conditions compared to other haplotypes (Figure 1f; Figure S3d). Remarkably, a strong negative correlation between *GmHE13* expression and hypocotyl length was observed (Figure 1g; Figure S3e), supporting GmHE13's role as a negative regulator of hypocotyl elongation.

**FIGURE 2:**
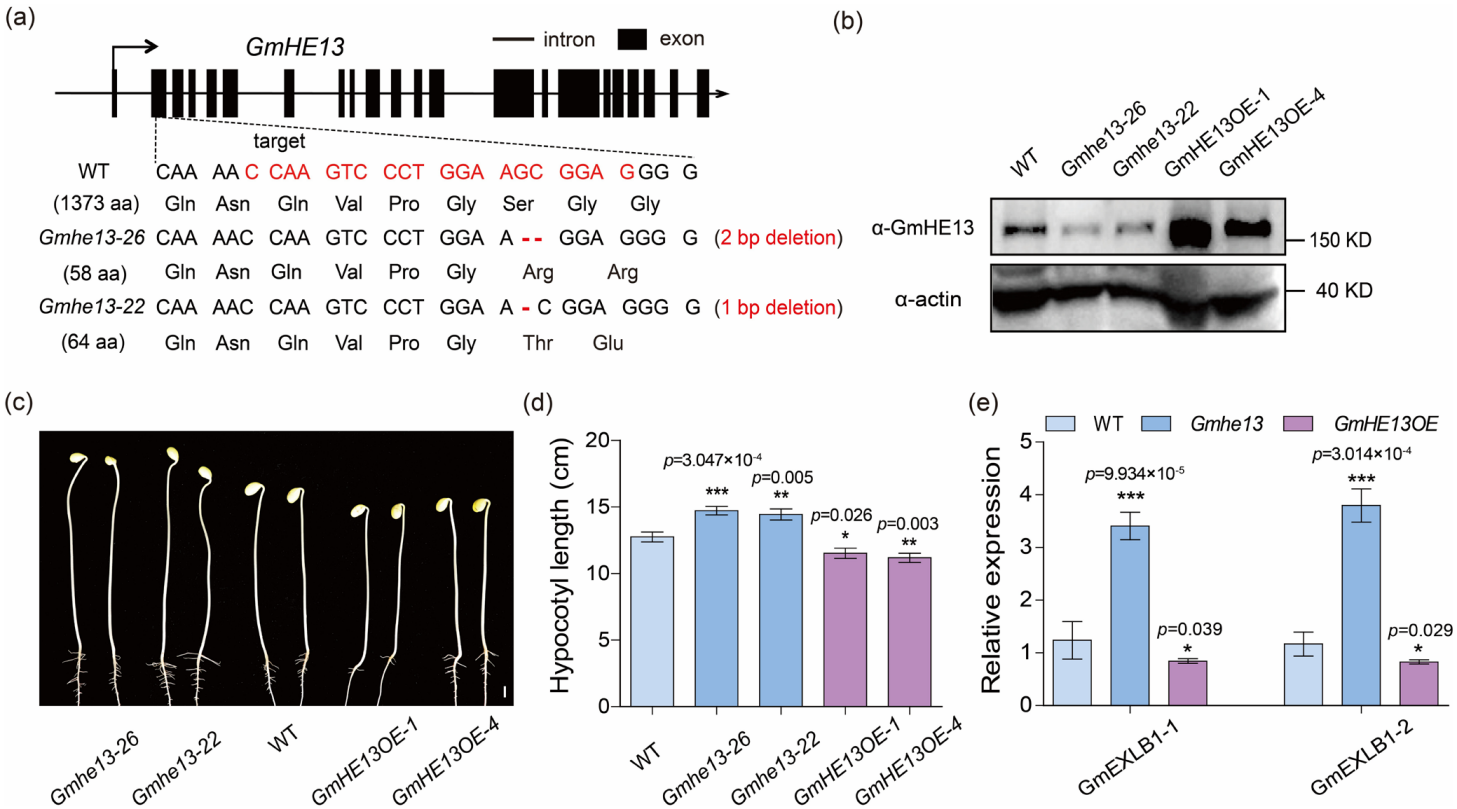
Functional analysis of *GmHE13* in altering hypocotyl elongation. (a) Schematic diagram showing the mutation sites in *Gmhe13* knockout mutants generated with CRISPR‐Cas9. *Gmhe13‐22* and *Gmhe13‐26* carry 1‐ and 2‐bp deletions, respectively, in the second exon of *GmHE13*. These frameshift mutations result in severely truncated proteins of 58 and 64 amino acids compared to the full‐length 1373‐aa protein. (b) Western blotting results showing diminished and elevated GmHE13 protein levels in *Gmhe13* and *GmHE13OE* hypocotyls, respectively, compared to that in WT (Wm82) control. (c) Representative photos of WT, *Gmhe13*, and *GmHE13OE* hypocotyls grown under dark conditions. Scale bar, 1 cm. (d) Quantification of hypocotyl lengths in (c). *n* ≥ 10. (e) RT‐qPCR results showing the elevated and reduced expression levels of *GmEXLB1‐1* and *GmEXLB1‐2* in *Gmhe13* and *GmHE13OE* hypocotyls, respectively. *n* = 3. Data represent mean ± SD. ***p < 0.001; **p < 0.01; *p < 0.05 (Student's *t*‐test).

RT‐qPCR analysis revealed that *GmHE13* expression was abundant in both green and dry soybean seeds but markedly reduced in hypocotyls and other post‐germination tissues (Figure S2c), consistent with its function as a negative regulator of post‐germination development. To elucidate GmHE13's role in hypocotyl elongation, we generated both overexpression and CRISPR/Cas9 knockout lines. We successfully obtained two knockout lines (*Gmhe13‐22* and *Gmhe13‐26*) containing 1‐ and 2‐bp deletions in the second exon of *GmHE13*, respectively. Both mutations result in severely truncated proteins (58 and 64 amino acids, respectively) compared to the full‐length 1373‐aa protein, leading to complete loss of function (Figure 2a). Additionally, we developed two overexpression lines (*GmHE13OE‐1* and *GmHE13OE‐4*) using the *GmUBI* (*Glyma.20G141600*) promoter. Western blot analysis confirmed significantly elevated GmHE13 protein levels in the overexpression lines and substantially reduced expression in the knockout lines (Figure 2b). After excluding potential confounding effects from differential germination rates (Figure S4a,b), we examined hypocotyl elongation in these transgenic lines under both light and dark conditions. Consistent with its proposed regulatory role, *Gmhe13* knockout lines exhibited significantly longer hypocotyls than wild type (WT) controls, whereas GmHE13 overexpression lines showed shorter hypocotyls (Figure 2c,d; Figure S4c,d). These results conclusively demonstrate that GmHE13 functions as a negative regulator of hypocotyl elongation in soybean. Of note, all genotypes (WT and mutant lines) displayed similar responsiveness to light inhibition (Figure S4e), suggesting that GmHE13‐mediated regulation of hypocotyl elongation operates independently of light signalling pathways.

### 2.3 GmHE13 Suppresses Histone Deacetylation on *Expansins*

To elucidate the mechanism underlying hypocotyl elongation regulated by GmHE13, we examined genes with altered expression in *GmHE13OE* hypocotyls through RNA‐seq. The results showed that overexpression of *GmHE13* resulted in a total of 418 differentially expressed genes (DEGs) in the hypocotyl (Table S3). Gene ontology (GO) analysis showed that the DEGs were enriched in biological processes such as ‘cell‐wall organisation’ and ‘plant‐type cell‐wall biogenesis’, etc. (Figure S4f). These DEGs include multiple *expansin* and *XET* genes (Figure S4g), which were reported to function in cell‐wall expansion and hypocotyl elongation (McQueen‐Mason et al. 1992; Wagstaff et al. 2010; Xu et al. 2016). Results of RT‐qPCR confirmed that the expression of two *expansin* genes, *GmEXLB1‐1* (*Glyma.05G065700*) and *GmEXLB1‐2* (*Glyma.17G147500*), were significantly downregulated in *GmHE13OE* hypocotyls and upregulated in *Gmhe13* hypocotyls (Figure 2e). Therefore, *GmHE13* may inhibit hypocotyl elongation by suppressing the expression of cell‐wall reorganisation genes including *expansins*.

*GmHE13* encodes a SNL family protein, containing a histone deacetylase interaction (HDI) domain. There are 12 members in the soybean SNL family. Phylogenetic analysis showed that the 12 GmSNLs were clustered into five groups (Figure S5a), in which GmHE13 was distributed at the same clade as AtSNL1/2 and another SNL, *GmHE19*. Remarkably, *GmHE19* was found in another LD block associated with hypocotyl elongation identified in SJZ replicate 1 (*p*‐value for lead SNP = 3.57 × 10^−6^, Figure S5b), indicating that GmSNLs play important roles in regulating hypocotyl elongation. Results of transient expression assays in tobacco (*Nicotiana benthamiana*) leaves showed that GmHE13‐CFP is expressed in the cytoplasm and the nucleus (Figure S6a), consistent with GmHE13's role as a potential histone modifying protein.

**FIGURE 3:**
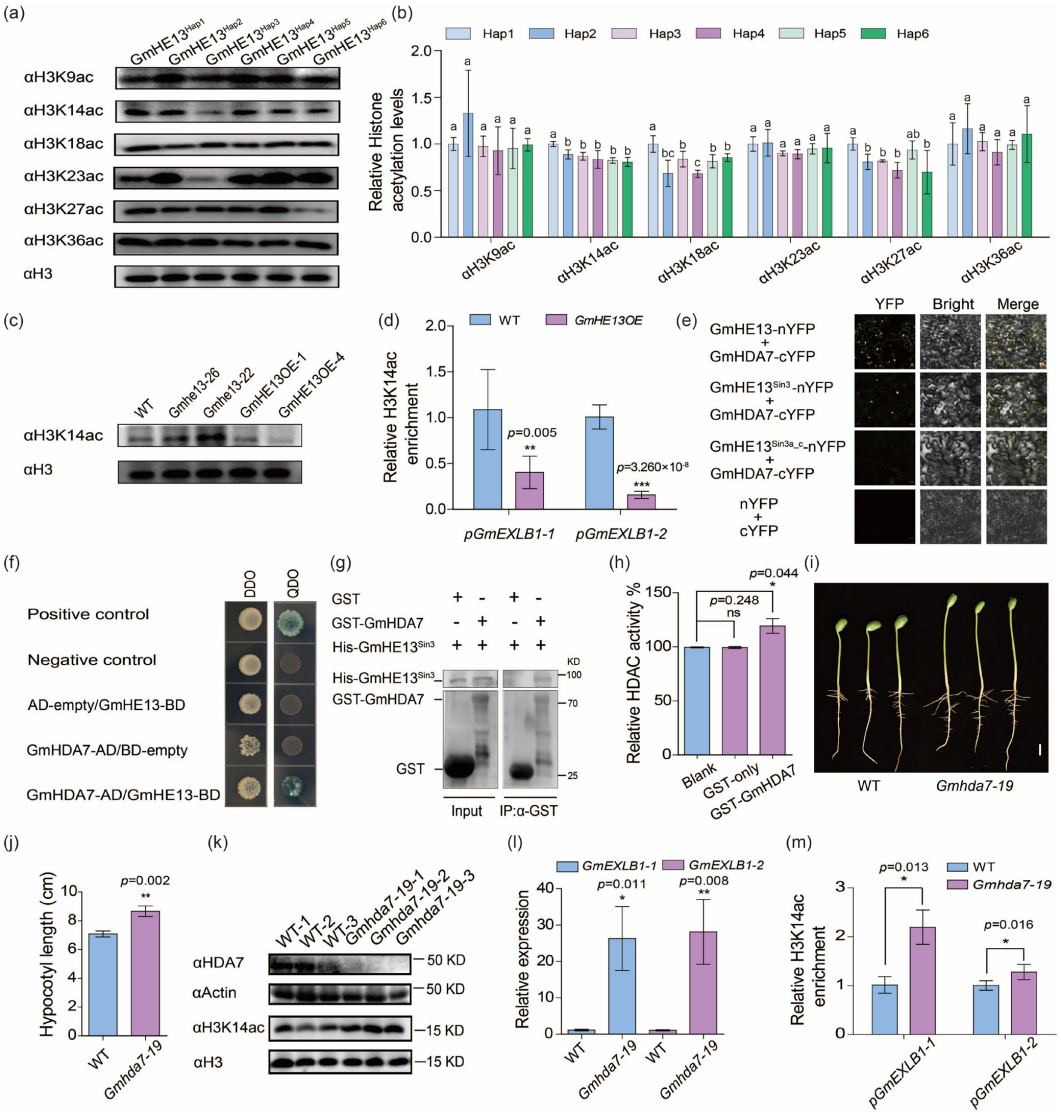
GmHE13 interacts with GmHDA7 to modulate histone acetylation and expression of target genes. (a) Representative western blotting results of global histone acetylation patterns (H3K9, H3K14, H3K18, H3K23, H3K27, and H3K36) in hypocotyls of soybean accessions carrying different *GmHE13* haplotypes. (b) Quantitative analysis of histone acetylation levels shown in (a). Data represent mean ± SD of three biological replicates. Significant differences among haplotypes were determined by one‐way ANOVA (*p* < 0.05), with different lowercase letters indicating statistically distinct groups. (c) Results of western blotting showing altered global H3K14 acetylation level in *Gmhe13* and *GmHE13OE* hypocotyls compared to that in WT (Wm82) control. (d) Results of CUT&RUN‐qPCR showing the reduced H3K14ac in the promoter regions of *GmEXLBs* within *GmHE13OE* hypocotyls. (e) Results of BiFC analysis showing the interaction between GmHE13 or GmHE13^Sin3^ and GmHDA7 in tobacco mesophyll cells. Scale bars, 20 μm. (f) Results of yeast two‐hybrid assays showing the interaction between GmHE13 and GmHDA7. Transformed yeast cells were grown on DDO (SD/−Trp‐Leu) or QDO (SD/−Trp‐Leu‐His‐Ade, with 4 mg/mL *α*‐X‐gal) medium. AD, GAL4 activation domain. BD, GAL4 DNA‐binding domain. (g) Results of pull‐down assays showing the direct interaction between purified His‐GmHE13^Sin3^ and GST‐GmHDA7. (h) Quantification of histone deacetylation activity of purified GST‐GmHDA7 protein with a HDAC activity assay kit. (i) Representative photos of WT and *Gmhda7‐19* hypocotyls. Scale bar, 1 cm. (j) Quantification of hypocotyl lengths in (i). (k) Western blotting results showing increased global H3K14ac level in *Gmhda7‐19* hypocotyls. (l) RT‐qPCR results showing the elevated expression of *GmEXLBs* in *Gmhda7‐19* hypocotyls. (m) Results of CUT&RUN‐qPCR showing the increased H3K14ac levels in the promoter regions of *GmEXLBs* within *Gmhda7‐19* hypocotyls. In (d), (h), (j), (l), and (m), data represent mean ± SD, with *n* = 3 in (d), (h), (l), (m) and *n* ≥ 10 in (j). ***p < 0.001, **p < 0.01, *p < 0.05 (Student's *t*‐test). ns indicates no significance.

To investigate GmHE13's potential role in histone deacetylation, we analysed global histone acetylation patterns in hypocotyls of different *GmHE13* haplotypes. Quantitative analyses revealed significantly elevated levels of active histone marks (H3K14ac, H3K18ac and H3K27ac) in GmHE13^Hap1^ hypocotyls compared to other haplotypes, with the exception of H3K27ac in GmHE13^Hap3^ (Figure 3a,b). These specific acetylation marks are well‐established epigenetic signatures of transcriptional activation in plants (Fang et al. 2014; Shen et al. 2019; Wu et al. 2023). Previous studies have demonstrated that altered expression of HDACs or their regulatory partners can differentially affect various histone acetylation marks (Benhamed et al. 2006; Guo et al. 2020; Mayer et al. 2019; Shen et al. 2019), supporting our hypothesis that GmHE13 functions as a key regulator of hypocotyl histone acetylation. Consistent with this model, we observed reciprocal regulation of H3K14ac levels in genetic manipulation experiments: *Gmhe13* lines showed significant hyperacetylation, while *GmHE13OE* hypocotyls exhibited reduced H3K14ac (Figure 3c). More importantly, CUT&RUN‐qPCR analysis demonstrated that *GmHE13* overexpression specifically decreased H3K14ac enrichment at the promoter regions of downstream targets *GmEXLB1‐1* and *GmEXLB1‐2* (Figure 3d). These findings establish a mechanistic link between GmHE13‐mediated histone deacetylation and transcriptional repression of *expansin* genes, revealing an epigenetic pathway regulating hypocotyl development.

### 2.4 GmHE13 Interacts With GmHDAs to Regulate Histone Acetylation of Target Genes

Studies across yeast, mammals, and plants have established that SNL proteins scaffold RPD3‐like histone deacetylases (HDACs) to form functional HDAC complexes (Asmamaw et al. 2024; Huang et al. 2019; Jing et al. 2021). Intriguingly, our previous co‐expression network analysis (Shen and Chen 2022) revealed that *GmHE13* shows coordinated expression with several *HDACs* and the histone acetyltransferase *GmGCN5* (*Glyma.19G160700*) in soybean hypocotyls, suggesting their potential involvement in a shared histone acetylation regulatory complex (Figure S6b,c). To investigate these relationships, we examined physical interactions between GmHE13 and four co‐expressed HDACs, GmHDA3, 7, 13, and 16. Bimolecular fluorescence complementation (BiFC) assays in tobacco leaves demonstrated robust interactions, with the HDI domain (GmHE13^Sin3^) proving both necessary and sufficient for these associations (Figure 3e; Figures S7b and S8a,b). Yeast two‐hybrid (Y2H) assays independently validated these interactions (Figure 3f; Figure S7c), with the exception of GmHDA16, which failed to show detectable interaction with GmHE13 in the yeast system. Finally, in vitro pull‐down assays confirmed direct physical binding between GmHE13 and GmHDAs (Figure 3g; Figure S8c). This comprehensive interaction analysis establishes GmHE13 as a core scaffold protein capable of assembling functional HDAC complexes in soybean.

The soybean genome encodes at least 15 HDACs, among which GmHDA3, GmHDA7, GmHDA13, and GmHDA16 are classified as type I RPD3‐like HDACs based on their conserved histone deacetylase domains (Figure S7a). Phylogenetic analysis reveals that GmHDA3, GmHDA7, and GmHDA16 cluster with *Arabidopsis* AtHDA6, a well‐characterised regulator of hypocotyl growth (Hao et al. 2016). Similarly, GmHDA13 groups with AtHDA9, which modulates hypocotyl elongation via its histone deacetylase activity (Kim et al. 2016). This phylogenetic conservation strongly suggests functional conservation in histone deacetylation‐mediated regulation of hypocotyl development. To biochemically validate this hypothesis, we performed in vitro deacetylase assays using purified GST‐tagged GmHDA proteins. Quantitative analyses demonstrated that all four GmHDAs (GmHDA3, 7, 13, and 16) exhibited dose‐ and time‐dependent deacetylase activity toward histone substrates (Figure 3h; Figure S9). Thus, these soybean HDACs possess functional histone deacetylase activity capable of modulating chromatin states at target loci.

To validate the role of GmHDAs in regulating gene expression and hypocotyl elongation, we examined the hypocotyl phenotype of an ethyl methanesulfonate (EMS)‐induced mutant line of *GmHDA7*, *Gmhda7‐19*, from the iSoybean mutant library (Figure S10a; Zhang et al. 2022). As anticipated, the truncated protein (Gmhda7^429aa^) caused by the mutation no longer interacts with GmHE13, as shown by the results of BiFC assays (Figure S10b). Phenotypic analysis indicated that while the seed germination rate was comparable to that of the WT (Figure S10c,d), the mutant hypocotyl lengths were significantly higher (Figure 3i,j). Consistent with this, the overall H3K14ac levels in *Gmhda7‐19* hypocotyls were significantly elevated (Figure 3k). Specifically, on the promoter regions of *GmEXLB1‐1* and *GmEXLB1‐2*, H3K14ac levels were upregulated, which further led to increased expression of *expansins* (Figure 3l,m).

Notably, our comprehensive interaction studies (Y2H, BiFC, and pull‐down assays) revealed that GmHE13 physically associates with the histone acetyltransferase GmGCN5 both in vivo and in vitro, with this interaction being mediated by its HDI domain (Figures S7b,c and S8b,c). This finding correlates well with their co‐expression pattern in hypocotyls (Figure S6b). As a well‐characterised HAT, GCN5 typically functions within multi‐protein complexes to promote transcriptional activation. In *Arabidopsis*, AtGCN5 acts as a negative regulator of hypocotyl elongation by activating light‐responsive genes (Benhamed et al. 2006). These results suggest a potential dual regulatory mechanism for GmHE13; while it scaffolds HDAC complexes to mediate histone deacetylation, it may simultaneously modulate GmGCN5's acetyltransferase activity through direct physical interaction. Such coordinated regulation would enable precise control of histone acetylation dynamics to fine‐tune the expression of downstream target genes.

### 2.5 GmRVE5 Guides the GmHE13‐HDAC Complex to Target Genes

**FIGURE 4:**
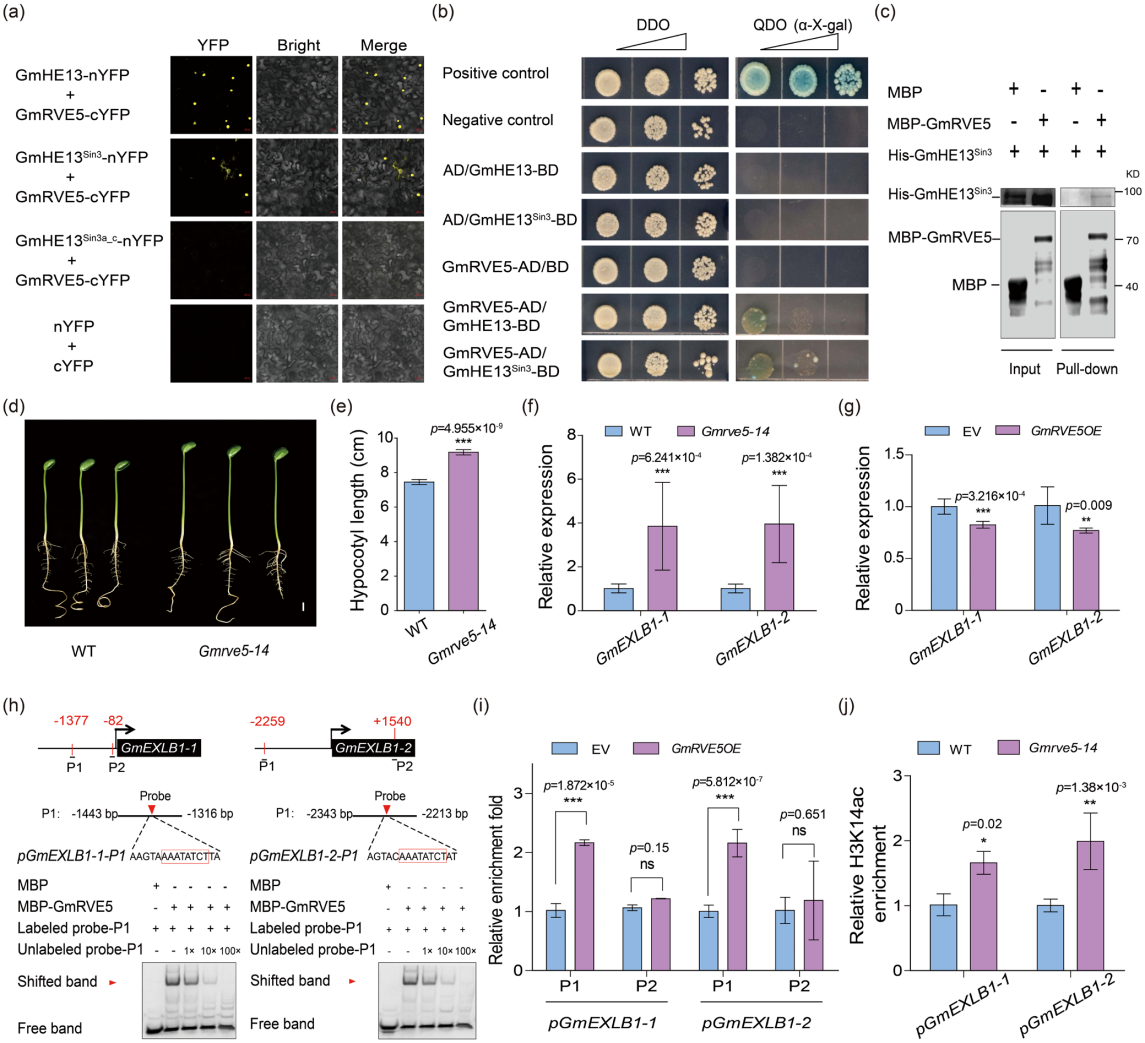
GmRVE5 guides the GmHE13‐HDAC complex to target genes. (a) Results of BiFC analysis showing the interaction between GmHE13 and GmRVE5 in tobacco mesophyll cells. Scale bars, 20 μm. (b) Results of yeast two‐hybrid assays showing the interaction between GmHE13 and GmRVE5 in yeast cells. *α*‐X‐gal was used as an indicator of a positive reaction at 4 mg/mL. The top triangles indicate a 10‐fold serial dilution with sterile water. (c) Result of pull‐down assay showing the interaction between purified MBP‐GmRVE5 and His‐GmHE13^Sin3^. (d) Representative photos of WT and *Gmrve5‐14* hypocotyls. Scale bar, 1 cm. (e) Quantification of hypocotyl lengths in (d). (f, g) RT‐qPCR results showing that the expression levels of *GmEXLBs* are upregulated in *Gmrve5‐14* hypocotyls (f) and downregulated in *GmRVE5OE* hairy roots (g). (h) Results of EMSA showing in vitro binding of GmRVE5 to the *GmEXLBs* promoter fragment containing the EE motif. (i) Results of CUT&RUN‐qPCR showing in vivo binding of GmRVE5‐GFP to *GmEXLB*s' promoter regions containing the EE motifs. (j) Results of CUT&RUN‐qPCR showing the increased H3K14ac levels in the promoter regions of *GmEXLBs* within *Gmrve5‐14* hypocotyls. Data represent mean ± SD, with *n* ≥ 10 in (e) and *n* = 3 in (f), (g), (i), (j). ***p < 0.001, **p < 0.01, *p < 0.05 (Student's *t*‐test). ns indicates no significance.

HDAC complexes typically include TFs to modulate acetylation levels at specific downstream gene sites (Gao et al. 2024; Jing et al. 2021; Zhao et al. 2019). To explore the TFs involved in the formation of GmHE13‐GmHDAC complexes, we analysed the cis‐elements in the promoters of the two *GmEXLBs* (2.5 kb from the TSS) and predicted potential TFs that bind to these motifs using PlantTFDB (https://planttfdb.gao‐lab.org/). We identified a total of 136 and 173 TFs with binding motifs in the promoter of *GmEXLB1‐1* and *GmEXLB1‐2*, respectively (Figure S11a; Table S4). Meanwhile, 28 TFs were predicted to be co‐expressed with *GmHE13* (Table S5). The intersection among the three TF sets includes only one member, a Myb‐like TF GmRVE5 (REVEILLE5, Glyma.16G032600) (Figure S11a). GmRVE5's homologue in *Arabidopsis*, AtRVE5, was reported as a transcriptional repressor in regulating thermoresponsive hypocotyl growth (Gray et al. 2017). Thus, we speculated that GmRVE5 is also a regulator of soybean hypocotyl elongation as a component of the HDAC complex. GmRVE5‐eCFP was shown to be localised in the nucleus of tobacco mesophyll cells (Figure S11b), consistent with its function as a TF. Through BiFC, we validated that GmRVE5 interacts with GmHE13 in tobacco leaves, and the HDI domain (GmHE13^Sin3^) is the core region for GmHE13's interaction with GmRVE5 (Figure 4a). Furthermore, through Y2H, we confirmed the interaction between GmHE13 and GmRVE5 in yeast cells (Figure 4b), while through pull‐down assays, we validated their direct interaction (Figure 4c). Notably, while we confirmed the interaction between GmHE13 and both GmRVE5 and GmHDA7, we failed to detect any direct physical association between GmRVE5 and GmHDA7 (Figure S11c). These results strongly suggest that GmHE13 functions as a scaffolding protein that physically bridges GmRVE5 and GmHDA7 to assemble a functional HDAC complex.

To investigate the function of GmRVE5 in hypocotyl development, we characterised an EMS‐induced mutant, *Gmrve5‐14* (Figure S11d). Phenotypic analysis revealed that while seed germination was unaffected in the mutant (Figure S11d,e), *Gmrve5‐14* exhibited significantly longer hypocotyls compared to WT plants (Figure 4d,e). Transcriptional profiling showed that expression levels of the two *expansin* genes, *GmEXLB1*‐1 and *GmEXLB1*‐2, were significantly elevated in *Gmrve5‐14* mutants (Figure 4f). Conversely, transient overexpression of *GmRVE5* in hairy roots led to marked downregulation of these target genes (Figure 4g), establishing GmRVE5 as a negative regulator of *expansin* gene expression.

Bioinformatic analysis identified one or more conserved evening elements (EE, 5′‐AAATATCT‐3′) in the promoters of both *GmEXLB* genes. Electrophoretic mobility shift assays (EMSA) demonstrated that recombinant MBP‐GmRVE5 protein specifically bound to the biotin‐labelled probe containing the EE motif (Figure 4h). CUT&RUN‐qPCR assays confirmed the in vivo binding in *GmRVE5‐GFP* overexpressing hairy roots, which showed significant enrichment of GmRVE5 at the EE‐containing regions of both *GmEXLB* promoters (Figure 4i). Further analysis revealed significantly higher levels of H3K14 acetylation at *GmEXLB* promoters in *Gmrve5‐14* mutants compared to WT plants (Figure 4j). Critically, EMSA assays revealed that GmHDA7 could only bind to the *GmEXLB* promoter fragments in the presence of both GmHE13 and GmRVE5, resulting in a supershift of the DNA‐protein complex (Figure S12). Since GmHDA7 does not directly interact with GmRVE5 (Figure S11c), these data demonstrate that GmHDA7 is recruited to the *GmEXLB* promoters via its association with GmHE13, which in turn bridges the interaction with GmRVE5. Together, these results establish GmHE13 as the essential scaffold that integrates GmRVE5 and GmHDA7 into a functional HDAC comple, whereby GmRVE5 recognises and binds target gene promoters, leading to histone deacetylation and transcriptional repression of downstream genes involved in hypocotyl elongation.

### 2.6 GmOBP3 Binds to *GmHE13* Promoter and Suppresses Its Expression

**FIGURE 5:**
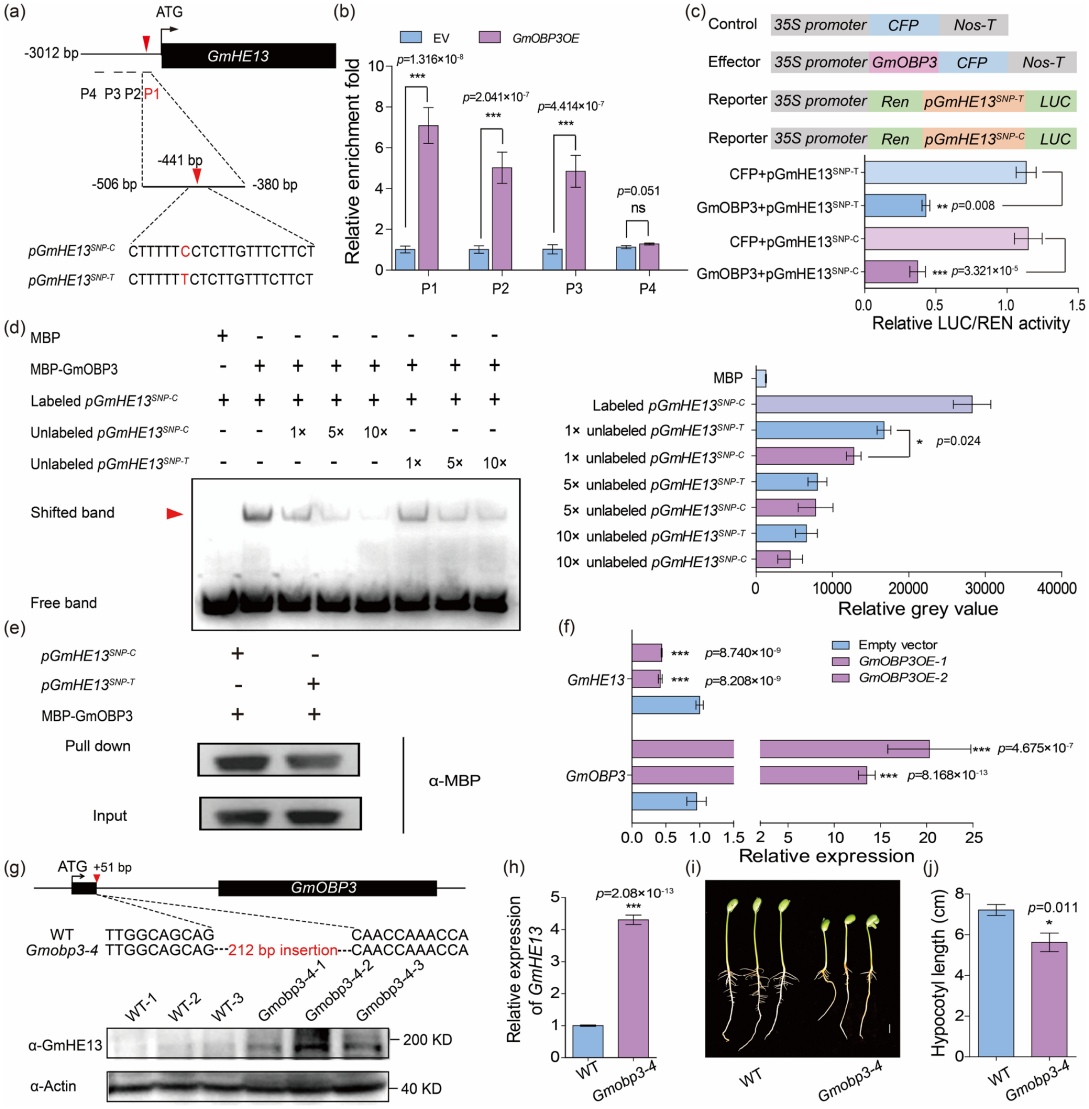
GmOBP3 binds to the *GmHE13* promoter and regulates hypocotyl elongation. (a) A potential binding motif of GmOBP3 (boxed) was found in the promoter of *GmHE13*. The red arrowheads and letters mark the lead SNP with two variants. P1–P4 marks the regions for qPCR quantification in (b). (b) Results of CUT&RUN‐qPCR showing the in vivo binding of GmOBP3 to the promoter of *GmHE13* in *GmOBP3OE* hairy roots. (c) Results of dual‐LUC assays showing the inhibited luciferase activity driven by the *GmHE13* promoter with different lead SNP variants (C or T). Schematic diagram on the top depicts the structure of the effector and reporter constructs. (d) Results of EMSA showing the differential interactions between GmOBP3 and *GmHE13* ^*SNP‐C*^ or *GmHE13* ^*SNP‐T*^ probes, *as* shown in (a). The right panel shows the quantification results of labelled probe levels (grey values) in the EMSA results. (e) Results of DNA pull‐down assay showing the differential binding affinity of GmOBP3 to *GmHE13* ^*SNP‐C*^ or *GmHE13* ^*SNP‐T*^ probes. (f) Results of RT‐qPCR showing the elevated expression of *GmOBP3* (below) and reduced expression of *GmHE13* (above) in *GmOBP3OE* hairy roots. (g) Western blotting result showing the increased GmHE13 levels in *Gmobp3‐4* hypocotyls. The top diagram shows that *Gmobp3‐4* mutant carrying a 212‐bp insertion in the 1st exon. (h) RT‐qPCR result of *GmHE13* expression level in *Gmobp3‐4* mutant line. (i) Representative photos of WT and *Gmobp3‐4* hypocotyls grown under light. Scale bar, 1 cm. (j) Quantification of hypocotyl lengths in (i). Data represent mean ± SD, with *n* = 3 in (b–d), (f), (h) and *n* ≥ 10 in (j). ***p < 0.001, **p < 0.01, *p < 0.05 (Student's *t*‐test). ns indicates no significance.

The lead SNP in *GmHE13* promoter is associated with its expression and hypocotyl lengths (Figure 1; Figure S3). One possible mechanism is that the SNP disrupts nearby cis‐regulatory elements, thereby affecting the binding activity of corresponding TFs. Therefore, we predicted the cis‐elements around the lead SNP and potential binding TFs (Figure S13a, Table S6). The results showed that the lead SNP is located inside a binding motif of the Dof3.6/OBP3 TF (5′‐CTTT/GTTTC‐3′), which was reported to act downstream of phyB to regulate hypocotyl elongation in *Arabidopsis* (Ward et al. 2005). There is only one Dof3.6/OBP3 homologue in soybean, Glyma.12G063800, which was designated as GmOBP3. We then hypothesised that GmOBP3 may directly bind to the *GmHE13* promoter, as the lead SNP (T>C) alters the core Dof3.6/OBP3 binding motif (5′‐CTTT/GTTTC‐3′ → 5′‐CTTT/GTTCC‐3′), potentially affecting protein‐DNA interaction and the regulatory effect. To verify this, we first performed CUT&RUN‐qPCR analysis in soybean hairy roots overexpressing *GmOBP3‐GFP*. The results demonstrated specific binding of GmOBP3 to the *GmHE13* promoter region containing the Dof3.6/OBP3 cis‐element (Figure 5a,b), confirming their physical interaction in planta. Next, dual‐luciferase (dual‐LUC) assays in tobacco leaves revealed that GmOBP3 functions as a transcriptional repressor of *GmHE13*, with slightly stronger inhibition observed for the *pGmHE13* ^*SNP‐C*^ variant compared to *pGmHE13* ^*SNP‐T*^ (Figure 5c). Furthermore, EMSA assays with recombinant MBP‐GmOBP3 protein demonstrated direct binding to both promoter variants and significantly stronger binding affinity for the *pGmHE13* ^*SNP‐C*^ variant, evidenced by reduced band intensity with *pGmHE13* ^*SNP‐C*^ competitor probes (Figure 5d). The allele‐specific repression suggested that the T>C SNP enhances GmOBP's regulatory effect. These findings were further corroborated by DNA‐protein pull‐down assays, which consistently showed preferential binding of GmOBP3 to the *pGmHE13* ^*SNP‐C*^ variant (Figure 5e). Thus, the base change of the lead SNP (T>C) indeed enhances the binding of GmOBP3 to the *GmHE13* promoter and causes greater inhibition of *GmHE13* expression.

The results of the transient expression experiment showed that GmOBP3‐GFP was expressed in the nucleus of tobacco mesophyll cells (Figure S13b), which is consistent with its function as a TF. To further elucidate the function of GmOBP3 in regulating the expression of *GmHE13*, we examined the expression of *GmHE13* in hairy roots overexpressing *GmOBP3* by RT‐qPCR. The results showed that overexpressed GmOBP3 significantly inhibited the expression levels of *GmHE13* (Figure 5f). To verify whether GmOBP3 is involved in the regulation of hypocotyl elongation, we examined the phenotype of a *GmOBP3* mutant line, *Gmobp3‐4* (Figure 5g; Zhang et al. 2022). Unsurprisingly, the hypocotyls of *Gmobp3‐4* were markedly shorter than the WT control (Figure 5i,j), while its seed germination speed was similar to WT (Figure S13c,d), confirming GmOBP3 as a positive regulator of hypocotyl elongation. Consistent with this, the results of RT‐qPCR and western blotting showed that the transcript and protein levels of *GmHE13* were both significantly increased in *Gmobp3‐4* hypocotyls (Figure 5g,h), further confirming that GmOBP3 acts as a negative regulator of *GmHE13* to control hypocotyl elongation.

### 2.7 *GmHE13* ^*Hap1*^ Has Undergone Artificial Selection

**FIGURE 6:**
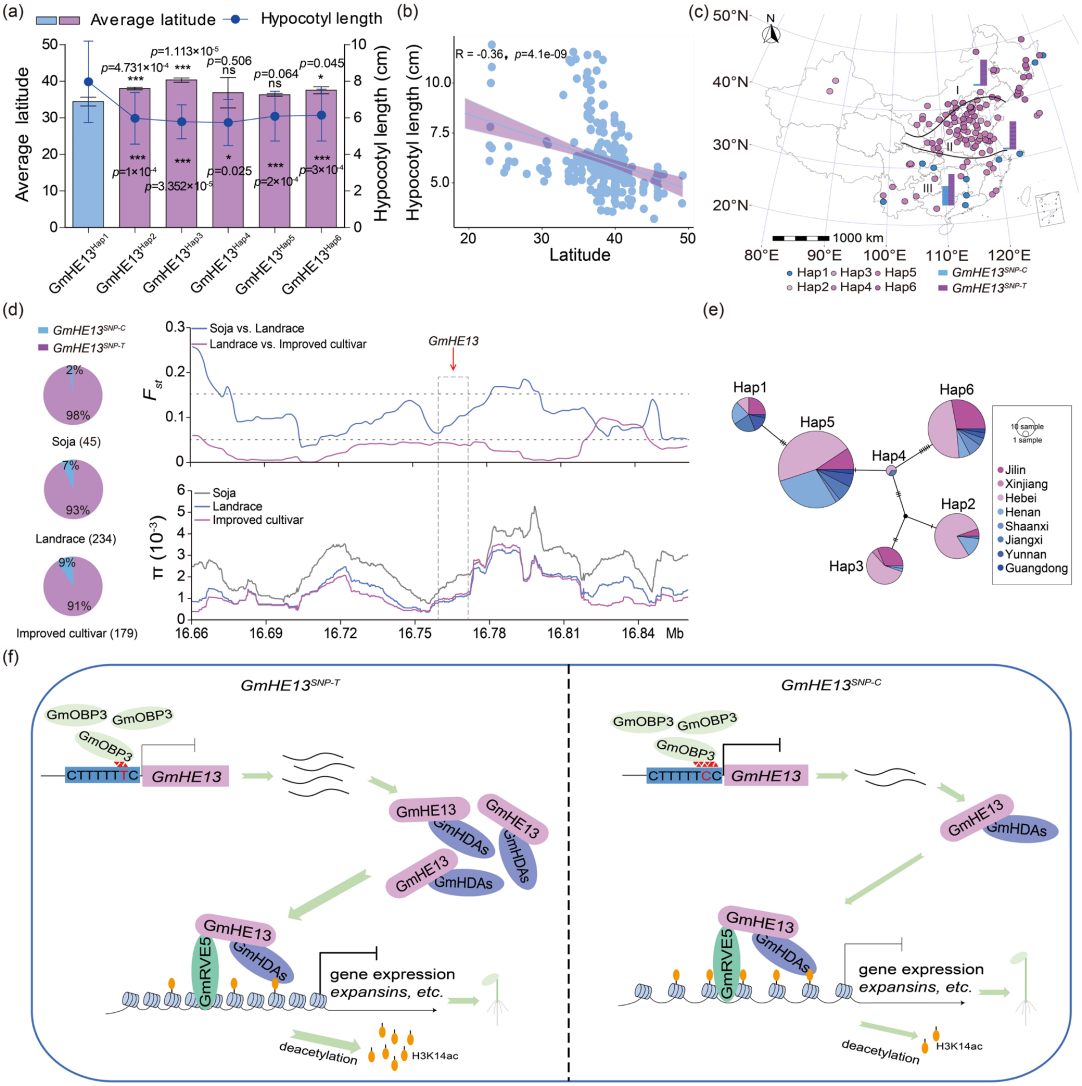
*GmHE13* ^*Hap1*^ was subject to selection during domestication. (a) Combo chart showing the differential hypocotyl lengths and latitudinal distribution of different *GmHE13* haplotypes. Hypocotyl lengths are displayed with line plot, while latitudinal distribution is displayed with histogram. (b) Negative correlation between hypocotyl length and native latitude of varieties in the soybean population. (c) Geographical distribution of *GmHE13* haplotypes around China, which are divided into three soybean‐growing regions with different latitudes. Histograms on the map indicates the proportion of *GmHE13* ^*SNP‐C*^ and *GmHE13* ^*SNP‐T*^ in different growing regions. (d) Population genetic analysis of GmHE13 across soybean germplasm groups. Left panel: Allele frequency distribution of the *GmHE13* ^*SNP‐T/C*^ among wild soybean ( *G. soja* , *n* = 45), landrace (*n* = 234), and improved cultivar (*n* = 179) accessions. Right panel: Genetic differentiation (*F*_st_) and nucleotide diversity (*π*) patterns across the GmHE13 genomic region. Values were calculated using a 20‐kb sliding window with 2‐kb steps. The dashed box highlights the *GmHE13* locus. Dotted lines indicate *F*_st_ thresholds of 0.05 and 0.15 (upper right inset). (e) Haplotype network generated on the basis of the maximum‐likelihood tree. Haplotypes are represented by circles whose size is proportional to the number of accessions carrying each haplotype. Vertical lines between circles represent mutations. Circles are coloured to match the distribution areas shown on the bottom left, where pink colours indicate high latitude areas and blue colours indicate low‐latitude areas. (f) A proposed working model summarising the function of GmHE13 in regulating soybean hypocotyl elongation. As a SNL protein, GmHE13 interacts with GmHDAs to form deacetylation complexes, which are recruited by GmRVE5 to the promoter of downstream genes such as *GmEXLB1‐1*/*2* and remove the H3K14 acetylation there, resulting in suppressed expression of *GmEXLBs* and shortened hypocotyls. *GmHE13* is bound and inhibited by a Dof TF, GmOBP3, while the binding affinity is differentiated via the lead SNP in the binding motif, causing differential expression of *GmHE13*, which further leads to variation in downstream gene expression and hypocotyl lengths. Data represent mean ± SD, with *n* ≥ 5. ***p < 0.001, *p < 0.05 (Student's *t*‐test). ns indicates no significance.

Our analyses reveal compelling evidence that *GmHE13* ^*Hap1*^, a haplotype associated with significantly longer hypocotyls (Figure 1d,e; Figure S3b,c), has undergone strong selection during soybean domestication and improvement. The geographical distribution of *GmHE13* haplotypes shows a striking latitudinal cline, with *GmHE13* ^*Hap1*^ predominantly found in low‐latitude regions (< 35° N) (Figure 6a) and a significant negative correlation shown between hypocotyl length and cultivation latitude (*r* = −0.36, *p* < 10^−8^, Figure 6b). This adaptive pattern is particularly evident in China's cultivation zones, where > 60% of *GmHE13* ^*Hap1*^ accessions are concentrated in southern regions (Figure 6c). Population genetic analyses reveal a clear selection signature, with *GmHE13* ^*Hap1*^ showing a progressive frequency increase from wild soybeans (2%) to landraces (7%) and modern cultivars (9%), implying its role in domestication and improvement. Besides, we observed moderate genetic differentiation (*F*_st_ = 0.05–0.15; Hartl and Clark 1997) between wild soybean ( *Glycine soja* ) and cultivated populations during initial domestication, along with a pronounced reduction in nucleotide diversity (*π*) in landraces and improved cultivars compared to wild progenitors (Figure 6d). These lines of evidence support that GmHE13^Hap1^ has undergone artificial selection during soybean domestication and breeding. The derived *GmHE13* ^*Hap1*^ likely originated from the ancestral *GmHE13^Hap4^* haplotype during soybean expansion southward (Figure 6e), with its long‐hypocotyl phenotype potentially conferring adaptive advantages in low‐latitude environments through improved light capture and seedling establishment, mirroring similar adaptations observed in *Arabidopsis* (Maloof et al. 2001; Stenøien et al. 2002).

## 3 Discussion

Hypocotyl elongation is crucial for seed‐seedling transition and post‐germination establishment. While extensively studied in *Arabidopsis* (Al‐Sady et al. 2006; Yu et al. 2013), species‐specific differences limit homology‐based translation of these findings to soybean. Our GWAS identified a significant LD block strongly associated with hypocotyl growth variation, with fine‐mapping pinpointing *GmHE13* as the causal gene (Figure 1). Functional characterisation established GmHE13 as a novel negative regulator of hypocotyl elongation (Figure 2; Figure S4). Intriguingly, this regulatory function appears to operate independently of light signalling pathways (Figure S4). This conserved photomorphogenic response across genotypes suggests that GmHE13 likely acts in an additive, rather than interactive, manner with canonical light signalling pathways. The functional significance of GmHE13 was further underscored by population genetic analyses revealing a cis‐regulatory polymorphism that creates two major haplotypes (Figures 1 and 5). The lead SNP alters a binding site for GmOBP3, a transcriptional activator of hypocotyl elongation (Figure 5; Figure S13). Mechanistically, GmOBP3 antagonises GmHE13 expression, creating a'derepressio' mechanism analogous to the well‐characterised COP1‐HY5 regulatory module in photomorphogenesis (Jing et al. 2021). This parallel suggests a conserved strategy for fine‐tuning hypocotyl growth in response to both developmental cues and environmental signals.

As an SNL protein, GmHE13 was shown to interact with GmHDA7 to form an SNL‐HDA complex that inhibits the expression of downstream genes through histone deacetylation, to regulate hypocotyl development (Figure 3). Other RPD3 HDACs interacting with GmHE13, as shown in our study (Figures S6–S8), may function through similar mechanisms, but we cannot exclude the possibility that they exhibit functional differentiation. Studies in *Arabidopsis* showed that different HDACs may have completely opposite regulatory effects. For example, HDA19 promotes hypocotyl elongation while HDA15 inhibits it, as they act on different target genes (Zhao et al. 2019; Jing et al. 2021). Furthermore, using H3K14ac as an example, we demonstrate how the HDAC complex scaffolded by GmHE13 modulates histone acetylation of downstream genes to regulate hypocotyl elongation (Figure 3). In addition to H3K14ac, other acetylation marks exhibiting haplotype‐specific patterns, including H3K18ac and H3K27ac (Figure 3), which are well‐established markers of gene activation as well (Benhamed et al. 2006; Fang et al. 2014; Shen et al. 2019; Wu et al. 2023), may likewise play important roles in different physiological contexts, warranting further investigation. A comprehensive analysis of the biological functions of these GmHDAs and their associations with various acetylation marks could provide valuable insights into the complex molecular network governing hypocotyl elongation. Notably, our results indicate that GmHE13, as an SNL scaffold protein, interacts with the HAT protein GmGCN5 as well (Figures S6 and S7), implying another feature of GmHE13 to modulate the histone acetylase activity of GmGCN5. Studies in mammals have demonstrated the association of HATs and HDACs in the same complex (Simone et al. 2004), so it is possible that GmGCN5 and GmHDAs exist in the same complex through the interaction with GmHE13, thereby balancing the histone acetylation levels of target genes.

The SIN3/SNL protein lacks intrinsic DNA‐binding activity and needs to be recruited to target DNA through interactions with TFs. In *Arabidopsis*, multiple TFs, such as HY5, have been reported to interact with HDACs and guide the HDAC complex to target genes (Jing et al. 2021; Zhao et al. 2019). Our data demonstrate that GmHE13 directly binds to GmHDA7 to form a complex, while GmRVE5, a MYB transcriptional repressor, physically associates with GmHE13, thereby guiding the complex to target genomic loci and modulating histone acetylation and expression of downstream genes, such as *GmEXLBs* (Figure 4; Figures S11 and S12). In this process, GmHE13 functions as a scaffold protein, a mechanism analogous to the mammalian Sin3‐HDAC complex, where Sin3 serves as a scaffold that recruits HDACs and non‐catalytic subunits, while TFs bind to Sin3 rather than directly to HDACs (Asmamaw et al. 2024). In contrast, studies in *Arabidopsis* have shown that HDA19 or HDA15 in the SNL‐HDA complex can directly interact with the TF HY5 (Jing et al. 2021; Zhao et al. 2019), suggesting potential divergence in HDAC complex architecture across species or functional contexts.

Our findings provide compelling evidence that the GmHE13‐scaffolded HDAC complex is recruited to the promoters of *GmEXLBs* (*expansins*), where it reduces histone acetylation and suppresses their expression (Figures 3 and 4). However, GmHE13 likely regulates additional targets beyond *GmEXLBs*, including genes involved in cell‐wall organisation (e.g., XETs) and oxidative stress responses (Figure S4). This hypothesis is supported by the observation that these genes are significantly altered in GmHE13 mutants (Table S3), and reactive oxygen species (ROS) accumulation during germination, a known regulator of seed dormancy release and cellular growth (El‐Maarouf‐Bouteau and Bailly 2008), which may further link these pathways to hypocotyl elongation. To further dissect this regulatory network, future studies employing knockout or overexpression of *GmEXLBs*, *XETs*, and other candidate targets could provide genetic evidence for GmHE13‐mediated hypocotyl elongation, offering deeper mechanistic insights.

In our proposed working model (Figure 6f), GmHE13 serves as a key regulatory hub that recruits HDACs and the TF GmRVE5 to form a repressive complex. This complex targets downstream genes involved in cell‐wall loosening, such as *expansins*, and suppresses their expression by removing H3K14 acylation, a histone modification associated with transcriptional activation. The consequent reduction in *expansin* expression ultimately leads to inhibition of hypocotyl elongation. The observed phenotypic variation between haplotypes may stem from the differential binding affinity of GmOBP3, a TF whose interaction with the *GmHE13* promoter is modulated by the lead SNP. This polymorphism alters *GmHE13* expression levels, thereby influencing the assembly and activity of the HDAC‐GmRVE5 repressive complex. Consequently, haplotypes with higher GmOBP3 binding affinity exhibit stronger *GmHE13* repression, leading to reduced HDAC recruitment and weaker suppression of hypocotyl elongation, while low‐affinity haplotypes result in the opposite effect. Our model provides a mechanistic link between genetic variation, epigenetic regulation, and phenotypic output, offering new insights into the molecular basis of soybean growth modulation.

## 4 Materials and Methods

### 4.1 Plant Materials and Growth Conditions

For the growth of the GWAS population, seeds of the 496 soybean ( *Glycine max* (L.) Merr.) accessions were grown in Shijiazhuang (114°52′ E and 38°05′ N), China, in 2018, with three biological replicates. For hypocotyl length measurement, the seeds were harvested and naturally air‐dried for 1 week before phenotyping. Detailed information of germplasm names and sources can be found in a previous study (Zhang et al. 2021). For phenotypic analysis of transgenic lines and mutants, soybean cultivar Williams 82 (Wm82) was used as the WT control. All the soybean plants used for phenotypic analysis were grown under short‐day conditions (12 h light/12 h dark) in a greenhouse with a light intensity of 500 μmol/m^2^/s at 25°C, 60% relative humidity. Tobacco plants used for subcellular localisation and BiFC analysis were grown in the same greenhouse.

### 4.2 Phenotypic Analysis of Soybean Hypocotyl Length

For soybean hypocotyl length measurement, uniform seeds were sanitised with chlorine gas for 24 h. Seeds were germinated under controlled conditions. After germination, 2‐days‐old seedlings were selected for uniform size and vigour and subsequently placed on sterilised vermiculite in a 0.9 × 0.6 × 0.13 m box, 20 seedlings per variety, spaced 0.5 cm apart. They were kept in a 25°C dark greenhouse for 7 days. Hypocotyl lengths were measured from the first lateral root to the cotyledon base. Genetic validation followed the same protocol in 10 cm square boxes.

### 4.3 GWAS Analysis of Hypocotyl Length in the Soybean Population

The kinship and population structure of the germplasm have been analysed in a previous study (Zhang et al. 2021). A total of 4 128 021 high‐quality SNPs and 1 166 033 InDels with a minor allele frequency (MAF) > 0.05 and a missing rate < 0.25 were used for this GWAS that has also been reported in a previous study (Zhang et al. 2021). Mean values (*n* ≥ 10) of the hypocotyl length in each soybean variety were calculated. Three biological replicates of the 18SJZ population were used for this analysis. GWAS was performed based on a MLM using the GAPIT software package (Tang et al. 2016). The threshold of the GWAS was set as −log_10_(*p*_*v*alue) = 7.92 based on the Bonferroni test threshold of −log_10_(0.05/total SNPs).

### 4.4 Haplotype Analysis

Haplotype analysis was performed by extracting all SNPs and InDels within the full‐length genomic regions of candidate genes identified through GWAS, including their 2.5 kb promoter regions. LD analysis, haplotype identification, and phenotype association analysis were then conducted using Haploview (Barrett et al. 2005). All variant information was obtained from the resequencing variant calling data of 496 soybean germplasm accessions (Zhang et al. 2021).

### 4.5 RNA Extraction and RT‐qPCR

Total RNA was extracted from at least 250 mg 7‐day‐old soybean hypocotyl using a plant RNA prep Kit (TianGen) according to the manufacturer's instructions. RNA concentration and purity were checked using the Qubit RNA Assay Kit by Qubit 2.0 Fluorometer (Life Technologies, CA, USA). First‐strand complementary DNA (cDNA) was synthesised with 2 μg total RNA using a Reverse Transcription kit (Vazyme) and 100 ng of first‐strand cDNA was used as the template for qPCR. The qPCR analyses were performed on a CFX96 Touch Real‐Time PCR Detection System (Bio‐Rad). The mRNA levels were calculated as described before (Chen and Penfield 2018). The reference gene was a soybean *ACTIN* gene (*Glyma.02G091900*). Three biological replicates with three technical replicates were performed for each sample. Primer sequences are listed in Table S7.

### 4.6 Vector Construction and Stable Plant Transformation

To generate the *Gmhe13* mutant using the CRISPR/Cas9 system, the CRISPR‐GE tool (http://skl.scau.edu.cn/) was utilised to design target sequences for *GmHE13*. The target‐gRNA elements were cloned into the *GmU6‐8* vector to form target‐gRNA expression cassettes, which were subsequently inserted into the *pCAMBIA1300‐Cas9* vector. These terminal constructs were transferred into the *Agrobacterium tumefaciens* strain *EHA105*. Stable transformations were performed in the Wm82 background using the cotyledon‐node method. To screen positive transformants, genomic DNA was extracted from the leaves of the T2 generation plants, and PCR was used to amplify the target sequences; then next‐generation sequencing analysis was employed to examine if the target sites were edited.

To construct the *GmHE13* overexpression plasmid, the full‐length coding sequence (CDS) of *GmHE13* was amplified and subsequently cloned into the *GmUbi‐3 × Flag‐Bar* vector. The construct was then introduced into *A. tumefaciens* strain *EHA105* and transformed into Wm82. Transgenic plants were screened using Basta. The GmHE13 protein levels in overexpression and CRISPR/Cas9 knockout lines were detected with a rabbit polyclonal GmHE13 antibody, which was generated by Abclonal. The homozygous overexpression lines and CRISPR/Cas9 knockout lines were selected and reproduced for at least four generations before being used for phenotypic observation.

*Gmrve5‐14*, *Gmhda7‐19*, and *Gmobp3‐4* mutant lines were obtained from the iSoybean mutant library (Zhang et al. 2022). All the mutant lines were backcrossed to Wm82 for three generations to purify the background before being used for phenotypic analysis.

### 4.7 Hairy Root Transformation

To construct the *GmOBP3* (*Glyma.12G063800*) and *GmRVE5* (*Glyma.16G032600*) overexpression plasmids, the full‐length CDS sequences were amplified from Ws82 and ligated into the *GmUbi‐GFP* vector. These two constructions and the empty vector were then transformed into *A. rhizogenes* strain K599 as described (Guo et al. 2011). The positive hairy roots were screened using a handheld fluorescence detector (LUYOR‐3415RG) and confirmed by RT‐qPCR analysis. At least three independent transgenic lines were used for gene quantification and to perform the CUT&RUN assay. The relevant primers are listed in Table S7.

### 4.8 RNA‐Seq Assay

RNA‐seq analyses were performed as described previously (Shen and Chen 2022). Briefly, at least 250 mg 7‐day‐old soybean seeding hypocotyls of Ws82 and *GmHE13‐OE* lines were collected for RNA extraction using a plant RNA prep Kit (TianGen). RNA integrity was assessed using the RNA Nano 6000 Assay Kit of the Bioanalyzer 2100 system (Agilent Technologies, CA, USA). Two biological replicates per line were used in RNA‐seq assays. Sequencing libraries were generated with fragmented mRNAs using the NEBNext Ultra RNA Library Prep Kit (NEB, MA, USA). The library preparations were sequenced on an Illumina Novaseq 6000 platform and 150 bp paired‐end reads were generated. The high‐quality clean reads were mapped to *Glycine max* V2.1 genome using Hisat2 with default parameters. Differential expression analysis was performed using the DESeq2 R package (1.42.1). Genes with an adjusted *p*‐value ≤ 0.05 found by DESeq2 were assigned as differentially expressed.

### 4.9 Subcellular Localisation

To generate *GmHE13‐GFP*, *GmOBP3‐GFP*, and *GmRVE5‐eCFP* vectors. Full‐length CDSs of the three genes were individually cloned and inserted into 35S‐GFP or pCAMBIA1300‐eCFP vectors. These constructions were then introduced into *N. benthamiana* by using an *A. tumefaciens* strain GV3101‐mediated transformation. After co‐culturing for 2 days under 16 h light/8 h dark conditions, tobacco leaves were snipped and visualised using confocal microscopy (Carl Zeiss LSM900). The excitation wavelength for GFP was 488 nm and for CFP was 405 nm. DAPI stain was used as a nucleus indicator. The relevant primers are listed in Table S7.

### 4.10 Transcription Factors and Cis‐Element Discovery Analyses

To identify the transcription factors (TFs) upstream of *GmHE13* and *GmEXLBs*, 2.5 kb sequences were extracted from these genes' promoter using the BioMart tool on the *EnsemblPlants* website (https://plants.ensembl.org/). These promoter sequences were then uploaded to the Binding Site Prediction tool on the PlantTFDB website (https://planttfdb.gao‐lab.org/) for scanning TFs upstream of the aforementioned genes (Jin et al. 2017). Additionally, cis‐motif discovery analysis was conducted by loading the promoter sequences into the MEME suite (https://meme‐suite.org/) with default parameters (Bailey et al. 2009).

### 4.11 Dual‐LUC Assay

Dual‐LUC assays were conducted as described previously (Wu et al. 2022). *GmHE13* promoter sequences, including the lead SNP, were amplified from Wm82 (*GmHE13* ^*SNP‐T*^) or Tianlong1 (*GmHE13* ^*SNP‐C*^), then cloned into the *pGreenII0800‐LUC* vector to generate reporters. The *35S:GmOBP3‐eCFP* was used as the effector. The reporters were co‐transformed with the effector or empty vector into tobacco leaves. After transformation, plants were grown at 22°C for 2 days before the luciferase activities were measured using a Dual Luciferase Reporter Gene Assay Kit (YEASEN). The relevant primers are listed in Table S7.

### 4.12 EMSA Assay

EMSA was performed using the LightShift EMSA Optimization & Control Kit (Thermo Fisher) according to the manufacturer's instructions with minor modifications. Briefly, to generate the biotin‐probes and cold‐probes of *pGmHE13*, two single‐strand probes at a concentration of 1 μM were used to generate a double‐stranded probe following a procedure that involved heating at 95°C for 5 min and then at 72°C for 20 min. 2 μg of purified MBP‐GmOBP3 protein was added to the binding reaction buffer and incubated for 10 min at room temperature (RT). Then, the labelled biotin‐probe, either individually or together with the cold‐probe, was added to the above reaction buffer and incubated for 20 min at RT. Subsequently, 5× loading buffer was added to terminate the reaction, and electrophoresis was performed on a 7% PAGE. The transferred Nylon membrane was exposed in a chemiluminescence apparatus (Tanon 5200). The GmRVE5‐*pGmEXLBs* EMSA assay was performed similarly. The relevant primers are listed in Table S7.

### 4.13 DNA Pull‐Down Assay

DNA Pull‐down was carried out as described previously, with minor modifications (Lyu et al. 2024). In brief, the aforementioned biotin‐labelled *pGmHE13* probes were separately incubated with the MBP‐GmOBP3 protein in a binding buffer for 30 min at RT, and then pulled down using streptavidin‐agarose beads. The combined abundance of the MBP‐GmOBP3 protein was detected by western blot assay.

### 4.14 CUT&RUN Assay

The nuclei were extracted from soybean hypocotyls and transgenic hairy roots using the cell lysis kit (Sigma) according to the manufacturer's instructions with minor modifications. In brief, 250 mg fresh samples were fully ground in liquid nitrogen and well mixed with 1 mL 1 × NIB buffer; then filtered using a 70 μm Cell Strainers (BD Falcon) and centrifuged at 1260 × *g* for 10 min under 4°C. The cell precipitate was suspended with 0.5 mL NIBA buffer and subsequently disintegrated with 0.25 mL 10% Triton X‐100 buffer. The cell lysate was slowly added into 0.8 mL 2.3 M Sucrose‐NIB buffer and waited for self‐settling. Finally, the cell lysate was centrifuged at 12 000 × *g* for 10 min at 4°C before the supernatant was removed, and the leftover was the nuclei.

The CUT&RUN assays were performed using the Hyperactive pG‐MNase CUT&RUN Assay Kit (Vazyme) according to the manufacturer's instructions. Briefly, the nuclei sample was washed with 500 μL washing buffer and resuspended in 100 μL washing buffer. 10 μL ConA Beads were then added, and the mix was incubated at RT for 10 min before being centrifuged to remove the supernatant. The nuclei‐beads complex was subsequently incubated with 1 μL antibody in 100 μL precooled antibody buffer at 4°C for 2 h. The sample was then washed twice with 800 μL Dig‐wash buffer and incubated in 100 μL pG‐MNase enzyme premixed solution at 4°C for 1 h. After washing with Dig‐wash buffer, 100 μL CaCl_2_ premixed solution was added to digest the nuclei‐beads complex on ice for 1 h. The reaction was finally stopped with 100 μL stop buffer containing spike‐in at 37°C for 30 min and centrifuged at 16 000 × *g* for 5 min at 4°C. The supernatant was transferred to a new tube and subsequently extracted using the FastPure Gel DNA Extraction Mini Kit (Vazyme) according to the product manual. The purified DNA was used to perform qPCR assays.

### 4.15 Histone Extraction and Western Blotting for Lysine Acetylation

Histones were extracted from at least 2 g fresh, 7‐days‐old hypocotyls for each sample, which were ground into powder in liquid nitrogen, then added with 20 mL precooled extraction buffer (Kim et al. 2016). The histones were precipitated with 1 g/mL trichloroacetic acid (TCA) and centrifuged at 17 000 × *g* for 30 min. The proteins were then washed with precooled acetone three times and subsequently air‐dried for 5 min. The histone powder was dissolved in 200 μL 1 × Laemmli buffer overnight and subsequently denatured at 98°C for 10 min. The histone acetylation levels were detected using western blotting. To ensure reproducibility, independent experiments were carried out at least three times. Histone modification levels were quantified by normalising the band intensity (grey value) of each modification against the corresponding histone H3 signal to control for potential loading variations. Quantitative analysis was performed using three biological replicates derived from the same haploid materials. The mean intensity values and standard deviations of histone modifications were calculated from these replicates to ensure statistical reliability.

### 4.16 BiFC Assays

For BiFC assays, the full‐length CDS, Sin3 domain, and Sin3a_c domain of *GmHE13* were amplified and cloned into *PXY‐104* vector, while the full‐length CDSs of *GmHDA3*, *GmHDA7*, *GmHDA13*, *GmHDA16*, *GmGCN5*, and *GmRVE5* were amplified and introduced into PXY‐106 vector. These *PXY‐104* and *PXY‐106* constructs were then co‐infiltrated into fully expanded young tobacco leaves in pairs. Infiltrated plants were grown at 25°C for 2 days before the fluorescent images were captured with a confocal microscope (Carl Zeiss LSM900).

### 4.17 Yeast Two‐Hybrid Assays

Yeast two‐hybrid assays were conducted according to a previous study (Wu et al. 2017). Briefly, the full‐length CDS of *GmHE13* fused with the DNA‐binding domain of GAL4 in the *pGBKT7* vector was used as bait. The full‐length CDS of *GmHDA3*, *GmHDA7*, *GmHDA13*, *GmHDA16*, *GmGCN5*, and *GmRVE5* was cloned into the *pGADT7* prey vector. *GmHE13‐BD* was co‐transformed with each prey vector and introduced into the Y2HGold strain. Positive transformations were screened in QDO with *α*‐X‐gal.

### 4.18 GST Pull‐Down

In vitro pull‐down assays were performed as described (Zhai et al. 2019). In brief, 20 μg of purified proteins of GST‐only, GST‐GmHDA3, GST‐GmHDA7, GST‐GmHDA13, GST‐GmHDA16, and GST‐GmGCN5 were individually incubated with 1 mL of GST binding buffer containing 60 μL of GST beads for 2 h at 4°C, and then washed twice in GST washing buffer. An equal volume of His‐GmHE13^Sin3^ domain recombinant protein was separately added to the above reaction and incubated in the GST pull‐down buffer for 2 h at 4°C. The bead‐protein complexes were eluted with a GST elution buffer for 30 min at 4°C and subsequently denatured at 98°C for 10 min. The pulled‐down products were detected using a western blot assay.

### 4.19 Phylogenetic Analysis and Sequence Alignment

For the phylogenetic analysis, the protein sequences of histone deacetylases in several species were extracted using TBtools from the peptide files that were downloaded from NCBI (https://www.ncbi.nlm.nih.gov/) (Chen et al. 2020). All the cleaned protein sequences were aligned, and the evolutionary relationships were constructed using the Neighbour‐Joining Tree method in MEGA7 software and subsequently modified in ITOL (https://itol.embl.de/) (Letunic and Bork 2021).

### 4.20 Histone Deacetylase Activity Analysis

The histone deacetylase activity was analysed using the histone deacetylase assay kit (Thermo Fisher), according to the manufacturer's instructions. About 10 μg purified GST‐fused HDAC proteins were added to the reaction buffer containing assay buffer and HDAC Substrate Solution, and incubated at 30°C for 30 min. 10 μL developer solution was then added to the reaction. After incubation at RT for 10 min, the fluorescence was measured using a fluorimeter plate reader (Tecan), with an excitation wavelength of 350–380 nm and an emission wavelength of 440–480 nm.

### 4.21 Statistical Analyses

Differences between the two samples were evaluated using two‐tailed Student's *t*‐test in Excel and GraphPad Prism 5.

## Author Contributions

M.C.: conceptualization, methodology, supervision, writing original draft, review and editing. Z.S.: investigation, data curation, formal analysis, writing original draft, review and editing. Wenqian Z., M.Y., Wei Z.: investigation, methodology. L.Z., F.L.: formal analysis, software, data curation (bioinformatics). Y.P.: methodology, resources (soybean transformation). P.Z., X.L.: investigation, validation (field work). W.W.: data analysis, visualisation.

## Conflicts of Interest

The authors declare no conflicts of interest.

## Supporting information


**Figures S1–S13:** pbi70310‐sup‐0001‐FiguresS1‐S13.docx.

**Tables S1–S7:** pbi70310‐sup‐0002‐TablesS1‐S7.xlsx.

## Data Availability

The RNA‐seq data that support the findings of this study are openly available in NCBI BioProject at https://www.ncbi.nlm.nih.gov/bioproject, reference number PRJNA1229468. Other data are available in this paper and its Supporting Information files.
