# Supplementary material for: The SNL Histone Deacetylase‐Binding Factor GmHE13 Is a Novel Regulator of Soybean Hypocotyl Elongation
Source: Plant Biotechnol J. 2025 Aug 12;23(12):5353–68. doi: 10.1111/pbi.70310 (PMC12665065; doi:10.1111/pbi.70310)
Supplement: Supplementary file 1 — Figures S1–S13: pbi70310‐sup‐0001‐FiguresS1‐S13.docx. [file PBI-23-5353-s001.docx]

**
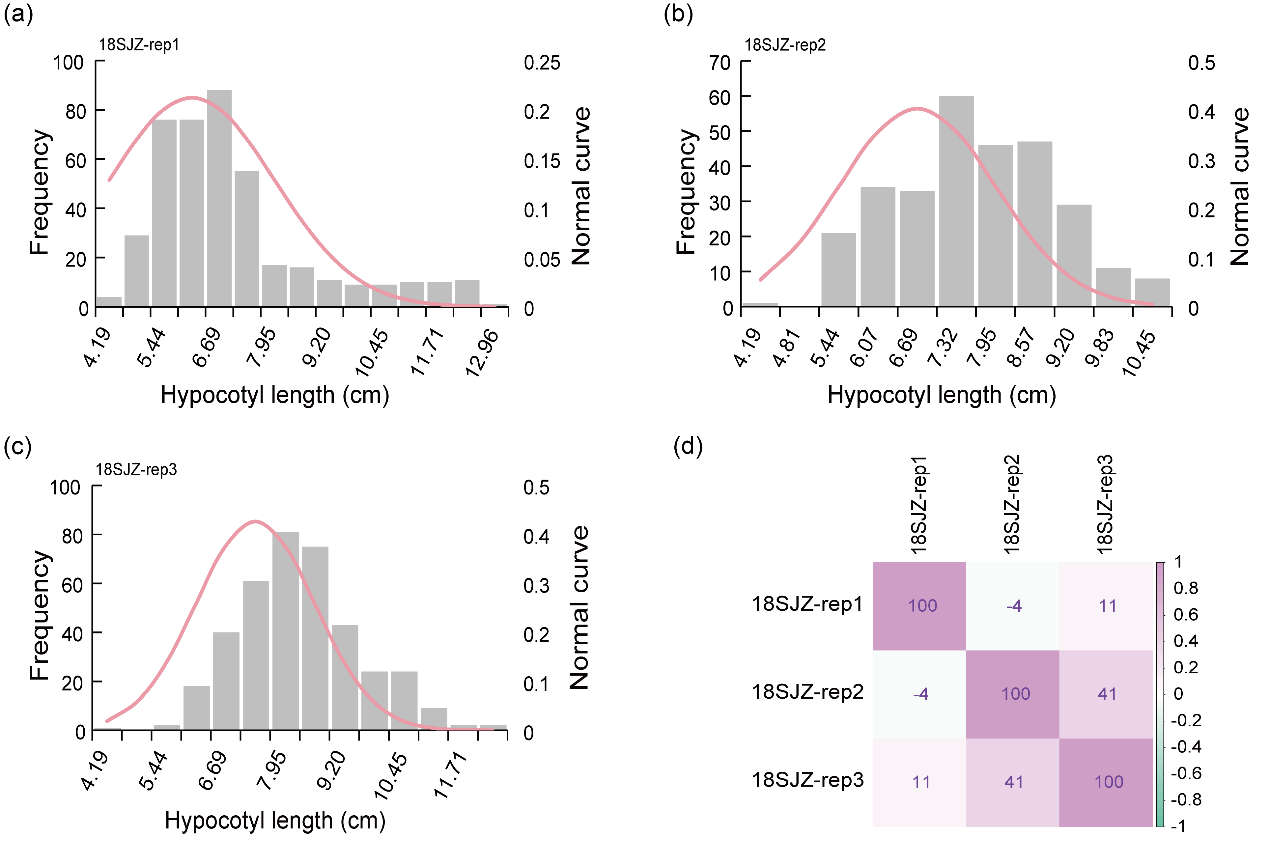
Figure S1 Phenotypic variation in hypocotyl lengths among soybean germplasms. (a-c)** Frequency distribution of hypocotyl lengths across three biological replicates of the 18SJZ population (soybean germplasm collection cultivated in Shijiazhuang, 2018). **(d)** Pairwise correlation matrix of hypocotyl measurements among replicates. Color gradient represents Pearson correlation coefficients: positive (pink), negative (green), and no correlation (white).


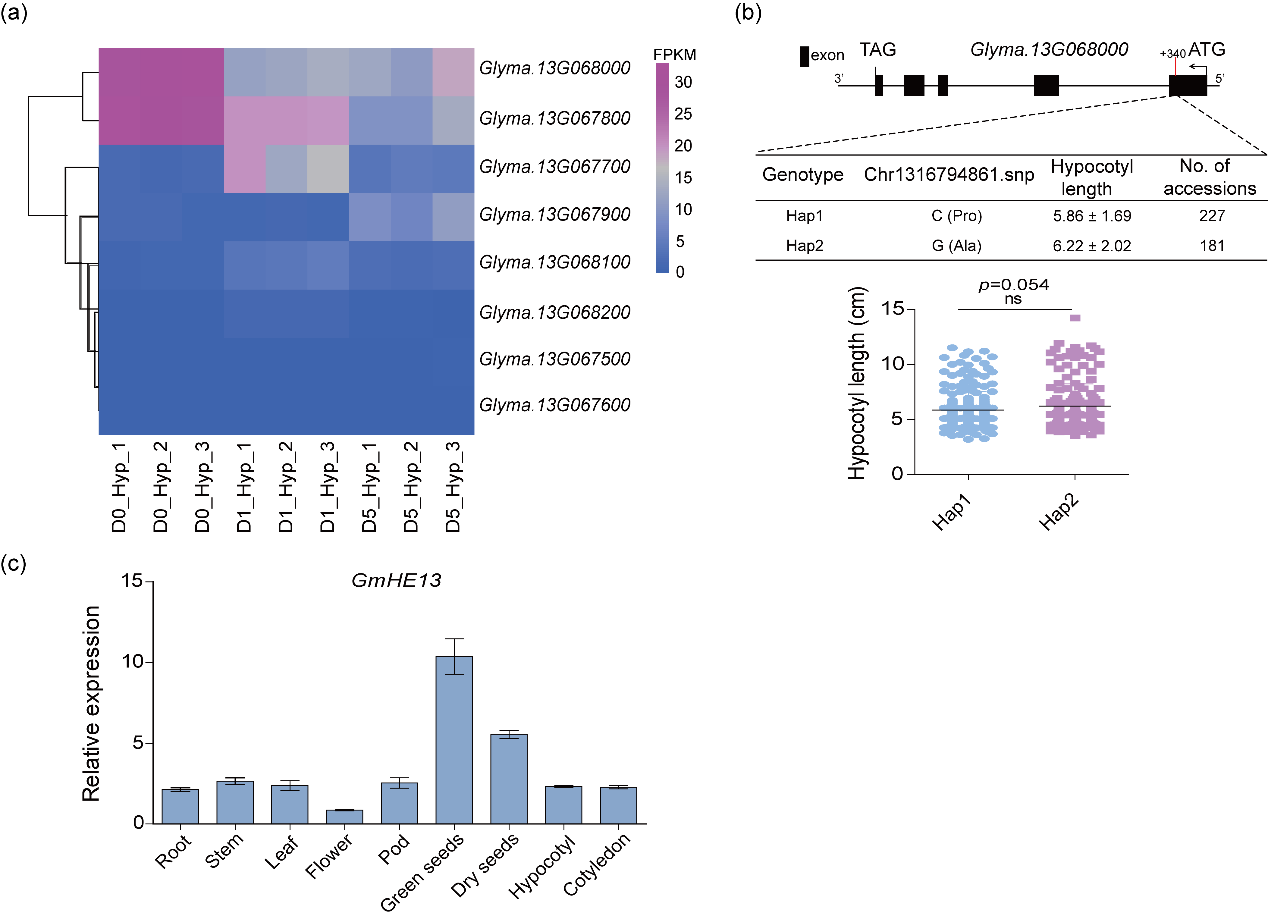


**Figure S2 Expression and haplotype analysis of candidate genes underlying the *qHE13* locus. (a)** Heatmap of *qHE13*-associated candidate gene expression levels. Expression values (FPKM, Fragments Per Kilobase of transcript per Million mapped reads) are depicted by a color gradient, with blue representing low expression and magenta indicating high expression. **(b)** Haplotype analysis of *Glyma.13G068000*. Top panel: genetic variation patterns defining the major haplotypes of *Glyma.13G068000*. Bottom panel: hypocotyl length comparison between the two predominant haplotypes. ns indicates no significance. **(c)** Tissue-specific expression profile (FPKM) of *GmHE13* across different soybean organs.


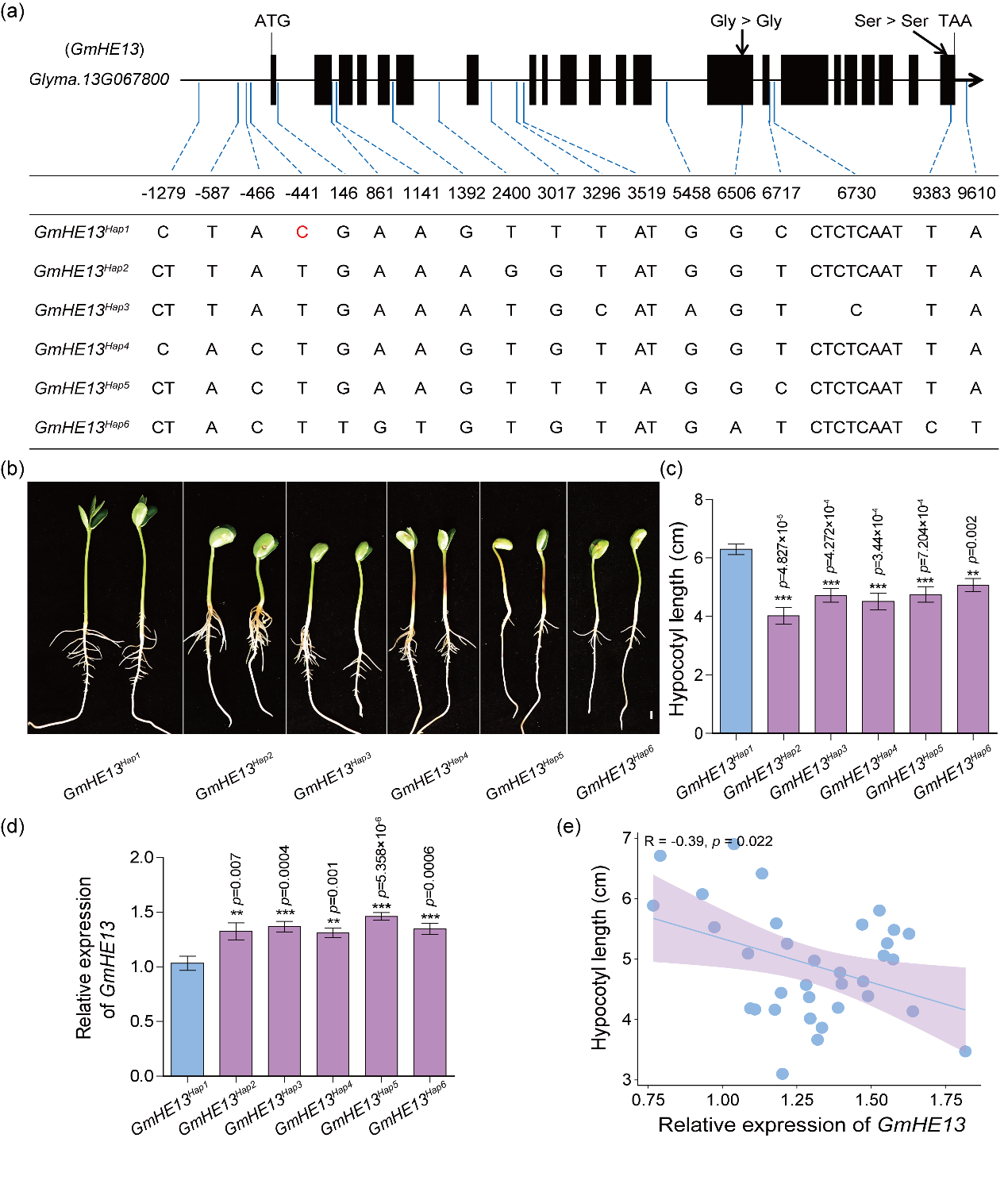


**Figure S3 Negative correlation between hypocotyl elongation and *GmHE13* expression levels.** **(a)** Haplotype structure of *GmHE13* based on 15 SNPs and 3 InDels. Physical positions of SNPs are indicated (top), with the lead SNP highlighted in red. Six major haplotypes were identified. **(b)** Representative images of hypocotyls from accessions carrying different *GmHE13* haplotypes grown under light conditions. Scale bars: 1 cm. **(c)** Hypocotyl length measurements for each haplotype (n = 9 randomly selected accessions per haplotype). **(d)** Relative *GmHE13* transcript levels in hypocotyls (RT-qPCR), showing significantly lower expression in *GmHE13^Hap1^* compared to other haplotypes. n = 3. **(e)** Negative correlation between hypocotyl length and *GmHE13* expression under light. Data represent mean ± SD. Statistical significance: *** *p* < 0.001, ** *p* < 0.01 (Student’s *t*-test).


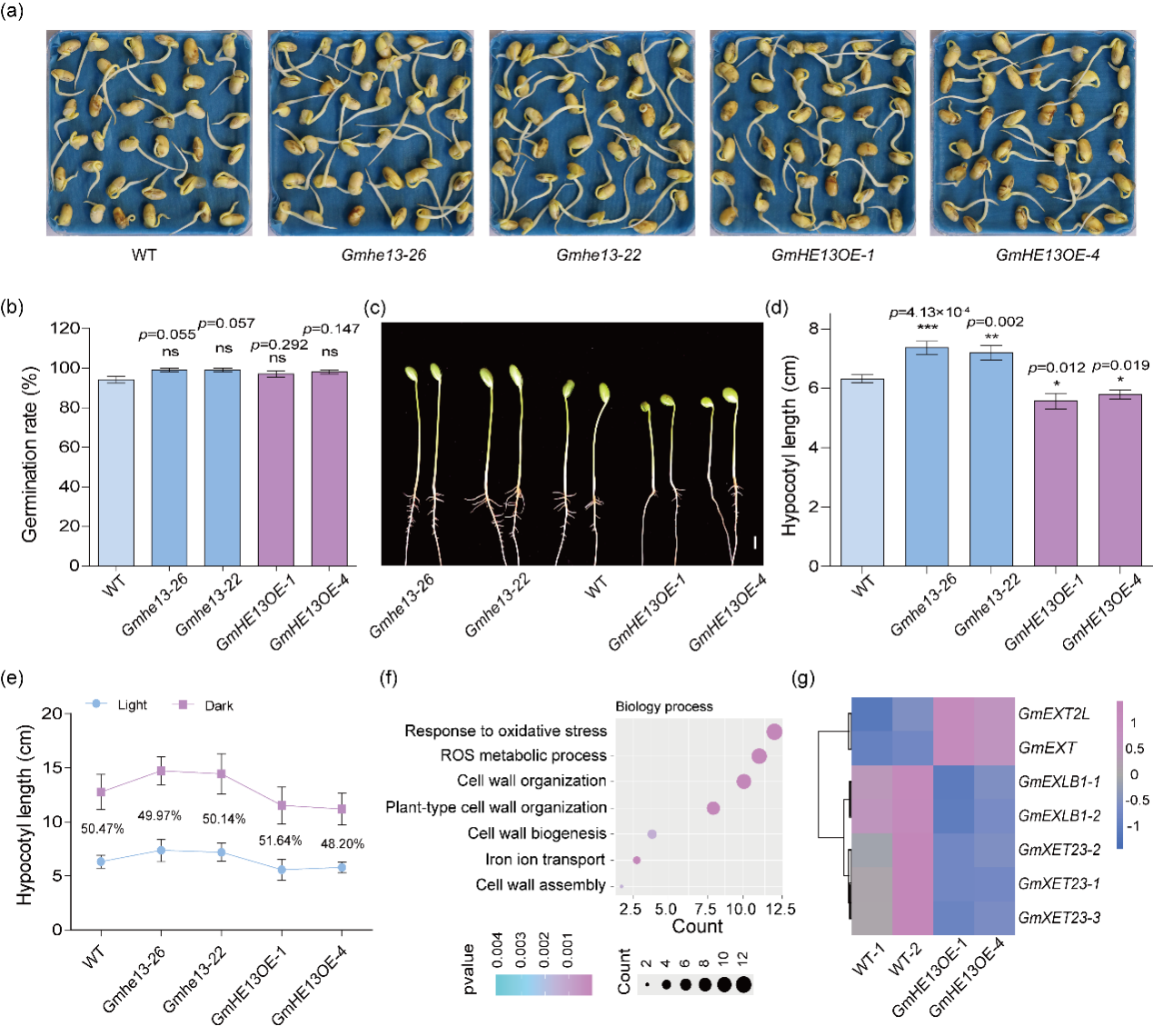


**Figure S4 Functional characterization of GmHE13 through overexpression and knockout analyses. (a)** Germination phenotypes of WT (Wm82), *Gmhe13* and *GmHE13OE* seeds at 48 hai (hours after imbibition) under dark conditions. **(b)** Germination rates of *Gmhe13* and *GmHE13OE* seeds at 48 hai compared to WT (n = 3 biological replicates, 36 seeds each). **(c)** Hypocotyl phenotypes of WT, *Gmhe13* and *GmHE13OE* seedlings grown under light. Scale bars: 1 cm. **(d)** Hypocotyl length measurements for genotypes shown in (c), with n ≥ 10 seedlings per line. **(e)** Relative reduction in hypocotyl length under light versus dark conditions for WT, *Gmhe13* and *GmHE13OE* lines. **(f)** GO enrichment analysis of DEGs in *GmHE13OE* hypocotyls. Dot size represents gene count while color indicates significance (*p* value). **(g)** Heatmap of selected DEGs (*expansins* and *XETs*) in *GmHE13OE* versus WT hypocotyls. Relative expression levels are scaled from low (blue) to high (pink). Data represent mean ± SD. Significance: ****p* < 0.001, ** *p* < 0.01, * *p* < 0.05 (Student’s *t*-test); ns, not significant.


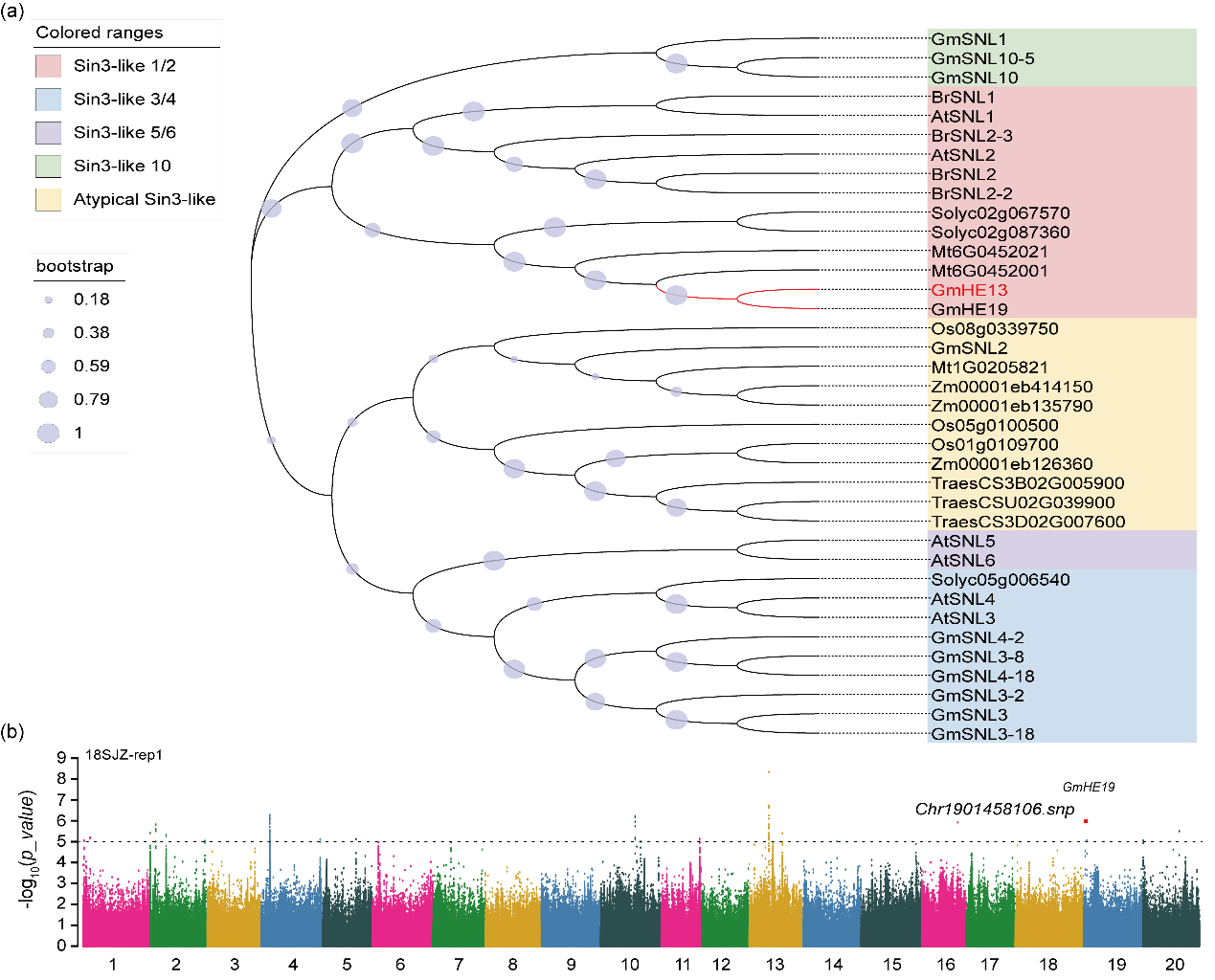


Figure S5 Phylogenetic analysis of soybean SNLs. (a) Bootstrapping phylogenetic tree of SNLs in Arabidopsis thaliana, Glycine max (soybean), Brassica rapa (oilseed rape), Oryza sativa (rice), Zea mays (maize), Triticum aestivum (wheat), Solanum lycopersicum (tomato), and Medicago truncatula. GmHE13 is in the clade marked red. The bootstrap values represent the confidence coefficients, as indicated by the size of the dots. (b) Manhattan plot showing GmHE19 as the candidate gene associated with hypocotyl elongation. The red dot indicates the lead SNP near GmHE19, while the dotted grey line indicates the significance threshold (*p* = 9.69 ×10^-6^).


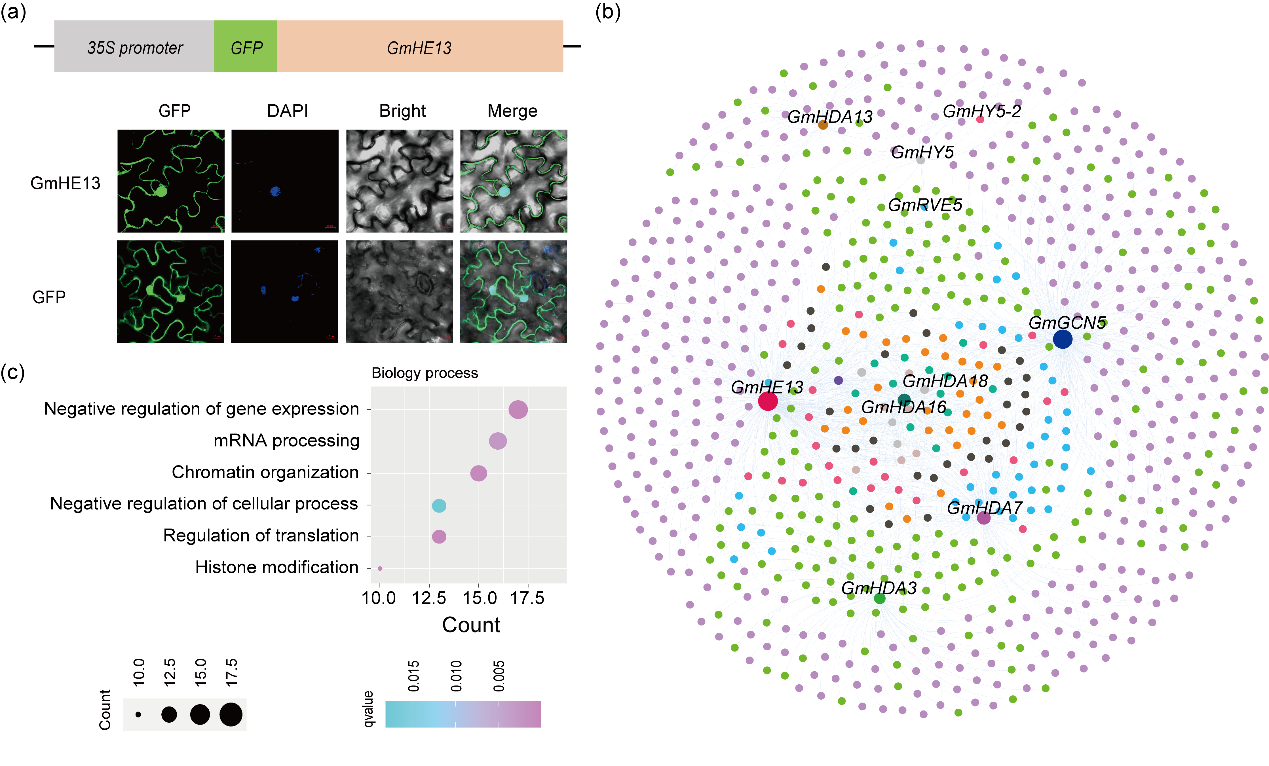


**Figure S6 Functional characterization of GmHE13 and its co-expression network.** **(a)** Subcellular localization of GFP-tagged GmHE13 in tobacco (*Nicotiana benthamiana*) mesophyll cells. DAPI staining indicates the location of the nuclei. Scale bars = 10 μm. **(b)** Weighted gene co-expression network analysis (WGCNA) identifying potential GmHE13 interactors. Key regulators of hypocotyl elongation (GmHDAs, GmGCN5, and GmHY5s) are highlighted. **(c)** GO enrichment analysis of GmHE13 co-expressed genes. Dot size represents the number of enriched genes, while color intensity indicates significance (*q-value*).


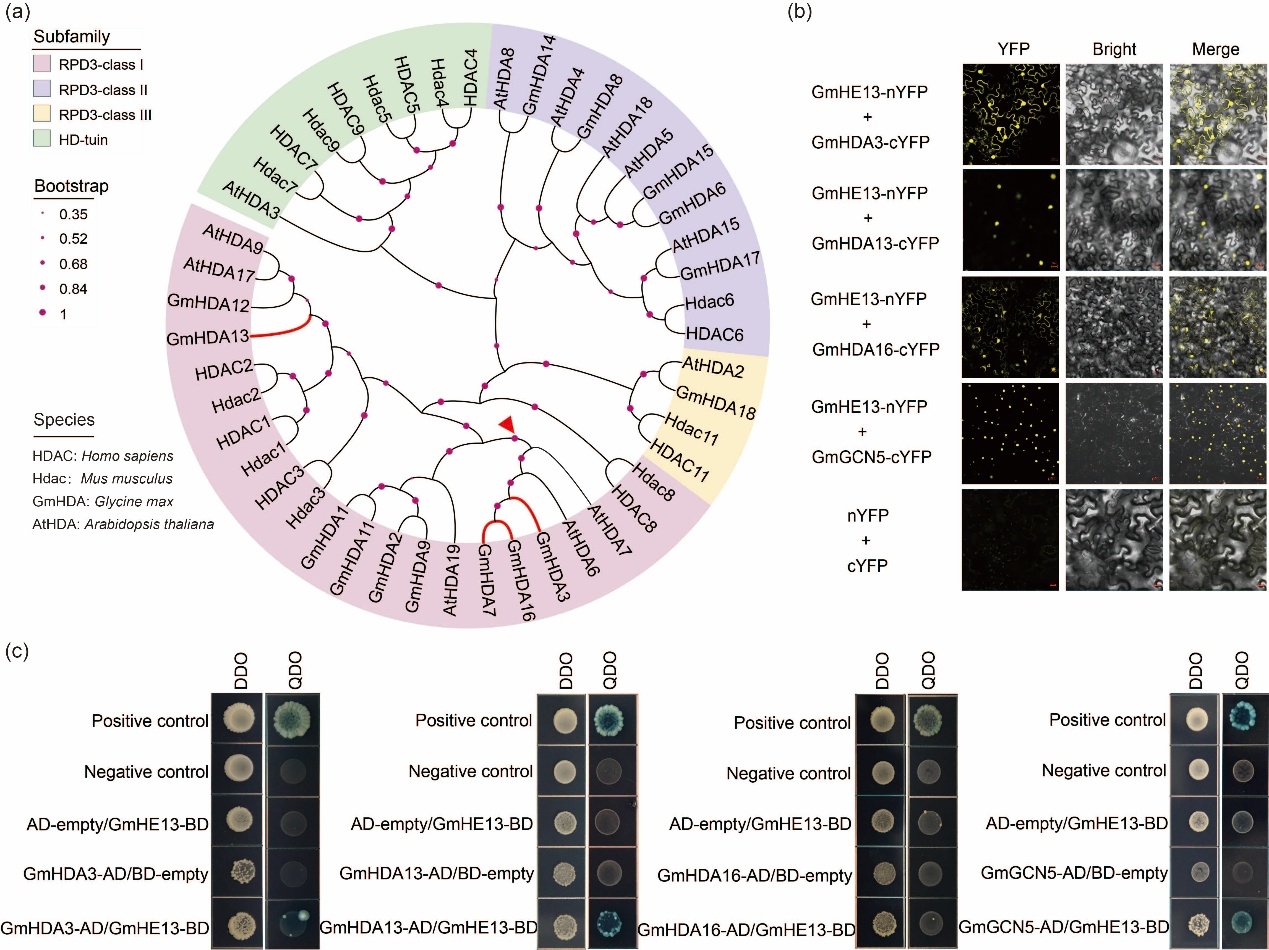


**Figure S7 Interactions between GmHE13 and GmHDAs/GmGCN5.** **(a)** Bootstrapping phylogenetic tree of HDACs in *Arabidopsis thaliana*, *Glycine max* (soybean), *Homo sapiens* (human) and *Mus musculus* (mouse). The bootstrap values represent the confidence coefficients, as indicated by the size of the dots. Red lines indicate the positions of GmHDA3, GmHDA7, GmHDA13 and GmHDA16， while red triangle indicates the subclade containing GmHDA3, GmHDA7 and GmHDA16. **(b)** Results of BiFC analysis showing that GmHE13 interacts with GmHDA3, GmHDA13, GmHDA16 and GmGCN5 in tobacco mesophyll cells. Empty vectors carrying nYFP or cYFP were used as negative controls. Scale bars, 20 μm (50 μm for fourth row). **(c)** Results of yeast two-hybrid assays showing that GmHE13 interacts with GmHDA3, GmHDA13, GmHDA16 and GmGCN5 in yeast cells. Transformed yeast cells are grown on DDO (SD/-Trp-Leu) or QDO (SD/-Trp-Leu-His-Ade, with *α*-X-gal) medium.


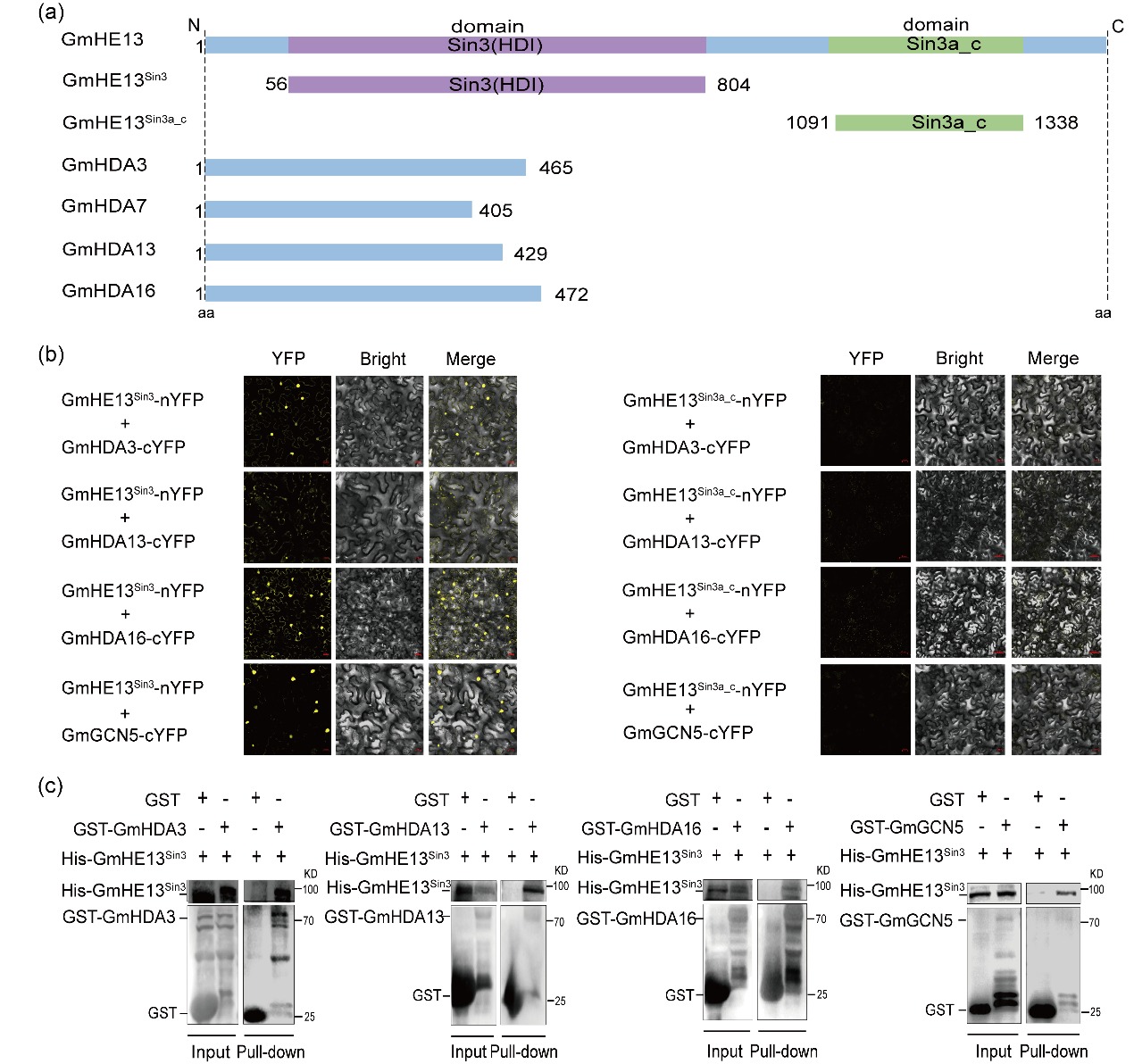


**Figure S8 Molecular interactions between GmHE13^Sin3^ and GmHDAs/GmGCN5.** **(a)** Schematic diagram showing the truncated GmHE13 proteins. GmHE13^Sin3^ represents the histone deacetylase interaction (HDI) domain, while GmHE13^Sin3a_c^ represents the GmHE13 without HDI domain. **(b)** Results of BiFC analysis showing that GmHE13^Sin3^, rather than GmHE13^Sin3a_c^, interacts with GmHDA3, GmHDA13, GmHDA16 and GmGCN5 in tobacco mesophyll cells. Empty vectors carrying nYFP or cYFP were used as negative controls. Scale bars, 20 μm. **(c)** Results of GST Pull-down assays showing that purified His-GmHE13^Sin3^ directly interacts with GST fused GmHDA3, GmHDA7, GmHDA16 and GmGCN5 in vitro.


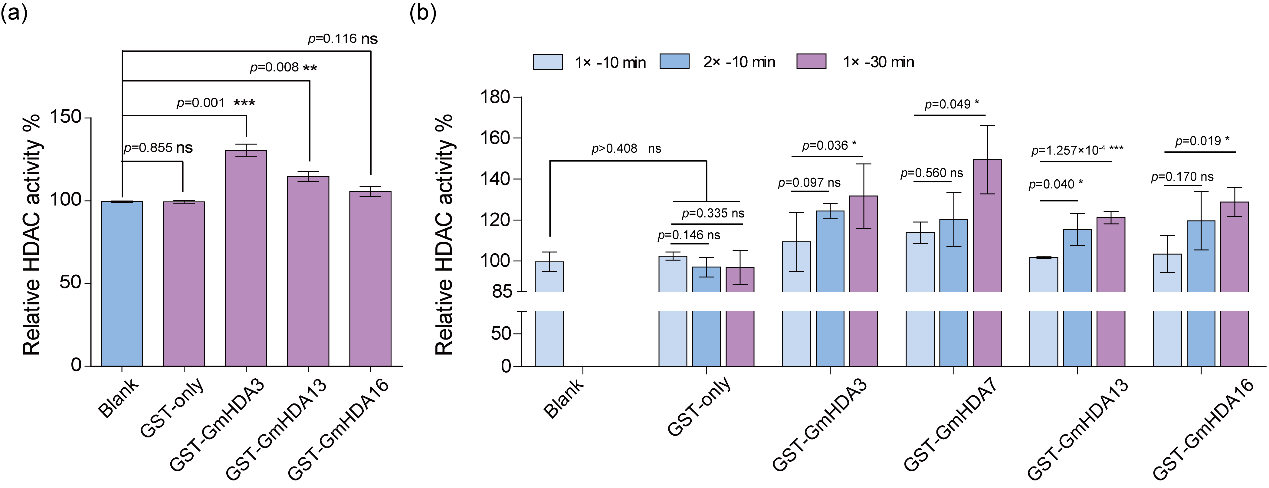


**Figure S9 Quantification of histone deacetylation activity of GmHDAs.** **(a)** Quantification of histone deacetylation activity of purified GST-GmHDAs with a HDAC activity assay kit. The empty GST vector was used as the control. **(b)** Quantification of histone deacetylation activity of GmHDAs, with different enzyme dosage or reaction time. Data are means ± SDs (n≥3). ***, *p* < 0.001, **, *p* < 0.01 (Student’s *t*-test). ns indicates no significance.


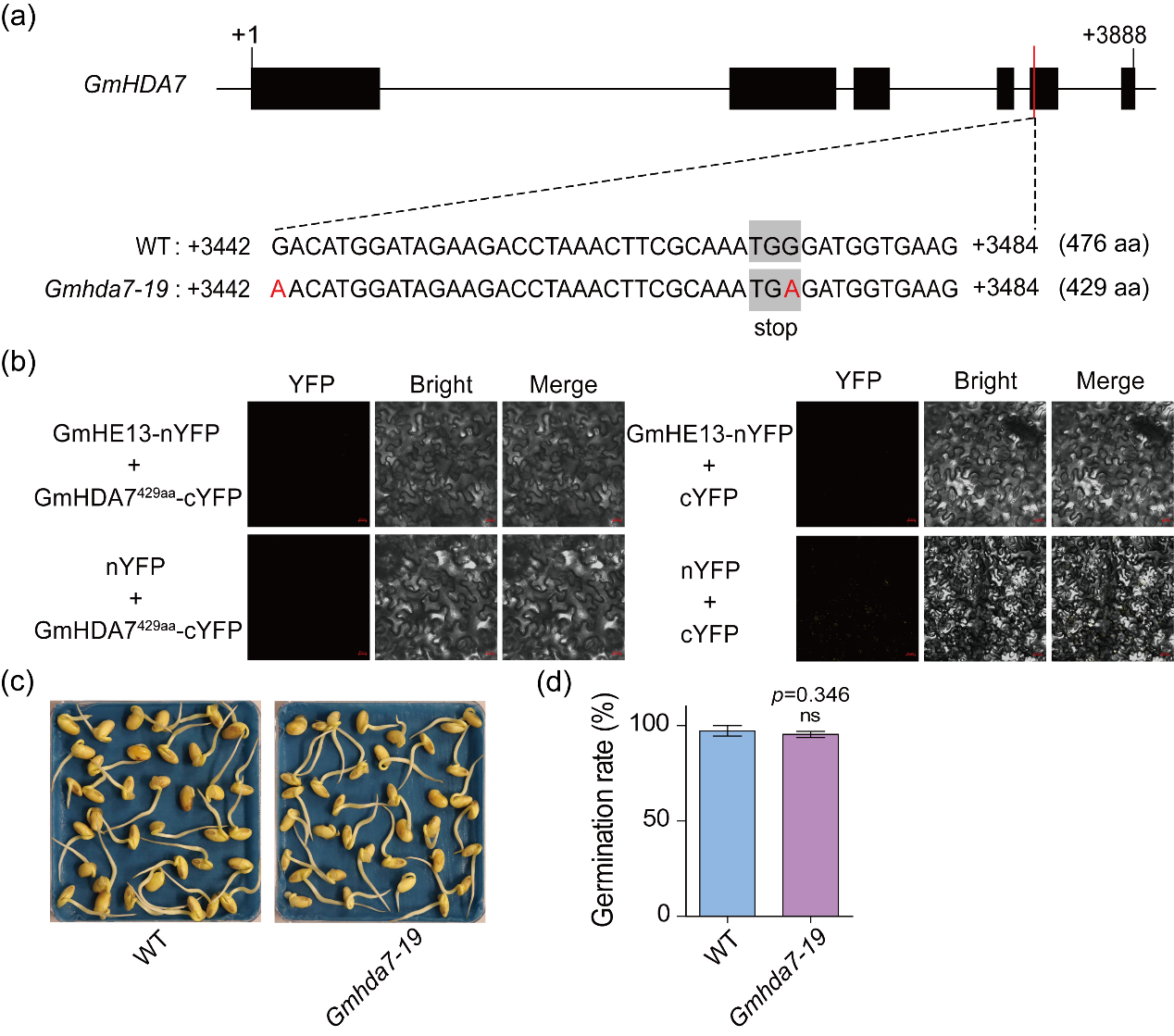


**Figure S10 Functional analysis of the *Gmhda7* mutant.** **(a)** *Gmhda7-19* mutant carries a single nucleotide mutation (G > A) causing premature termination. **(b)** Results of BiFC analysis showing that the interaction between GmHE13 and GmHDA7 was abolished by the *Gmhda7-19* mutation. Scale bars, 20 μm (50 μm for sencond row on the right). **(c)** Representative photos of WT and *Gmhda7-19* mutant seed germination at 48 hai under dark conditions. **(d)** Germination rates in (c), n = 3 biological replicates, with 36 seeds per replicate. Statistical significance was determined using Student's *t*-test. ns indicates no significance (*p* > 0.05).


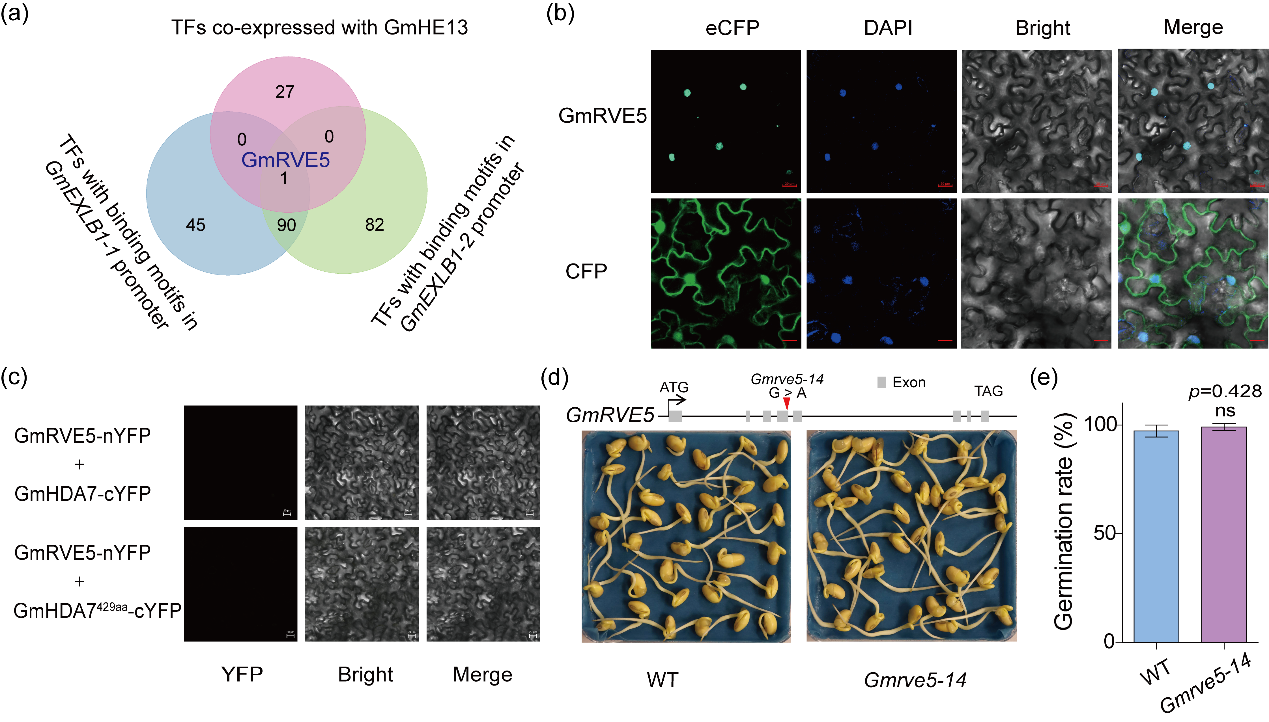


**Figure S11 Prediction of GmRVE5 as a TF interacting with GmHE13 to regulate the expression of *GmEXLBs.*** **(a)** Venn diagram showing GmRVE5 as the only common member among TFs co-expressed with GmHE13 and TFs that with binding motifs in the promoter of *GmEXLB1-1/2*. **(b)** Subcellular localization of GmRVE5-CFP in tobacco mesophyll cells. DAPI staining indicates the location of the nuclei. Scale bars, 20 μm. **(c)** BiFC analysis demonstrating the absence of interaction between GmRVE5 and GmHDA7 in tobacco leaf cells. Scale bars, 20 μm. **(d)** Representative photos of WT and *Gmrve5-14* mutant seed germination at 48 hai under dark conditions. Top panel: schematic illustrating the mutation position of *Gmrve5-14* relative to *GmRVE5* gene structure elements (exons, introns, and UTRs). **(e)** Germination rates in (d), n = 3 biological replicates, with 36 seeds per replicate. Statistical significance was determined using Student's *t*-test. ns indicates no significance (*p* > 0.05).


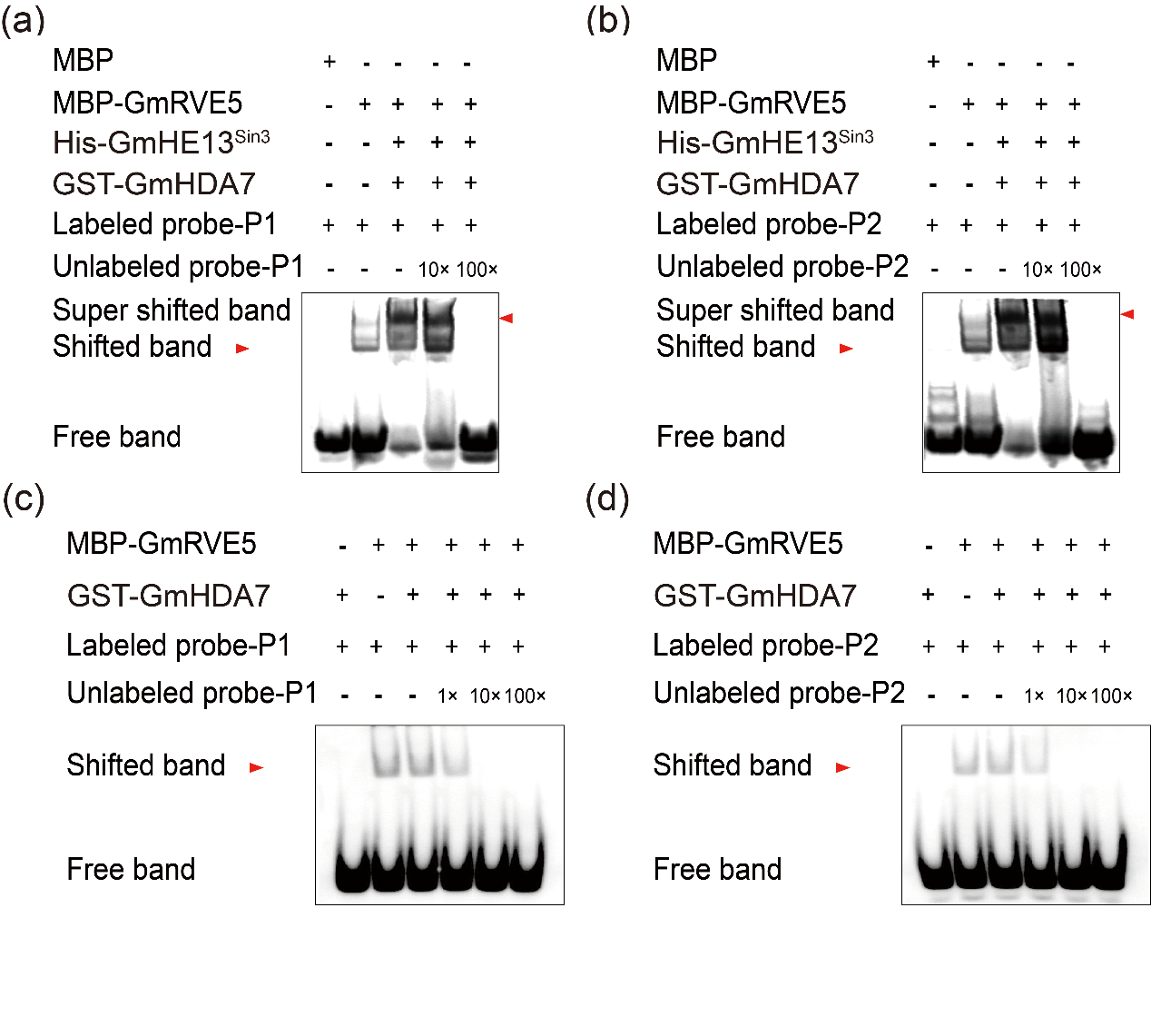


**Figure S12 GmHDA7 depend on GmHE13 and GmRVE5 to bind to the *GmEXLB* promoters.** **(a, b)** EMSA results demonstrating that the addition of His-GmHE13^Sin3^ and GST-GmHDA7 induces a supershift of the protein-DNA complex formed by MBP-GmRVE5 with the promoter fragments of *GmEXLB1* (a) or *GmEXLB2* (b). **(c, d)** EMSA results showing that GST-GmHDA7 alone cannot bind to the *GmEXLB* promoter fragments. Moreover, in the absence of GmHE13, GST-GmHDA7 fails to induce a supershift of the MBP-GmRVE5–promoter complex, even when MBP-GmRVE5 is present.


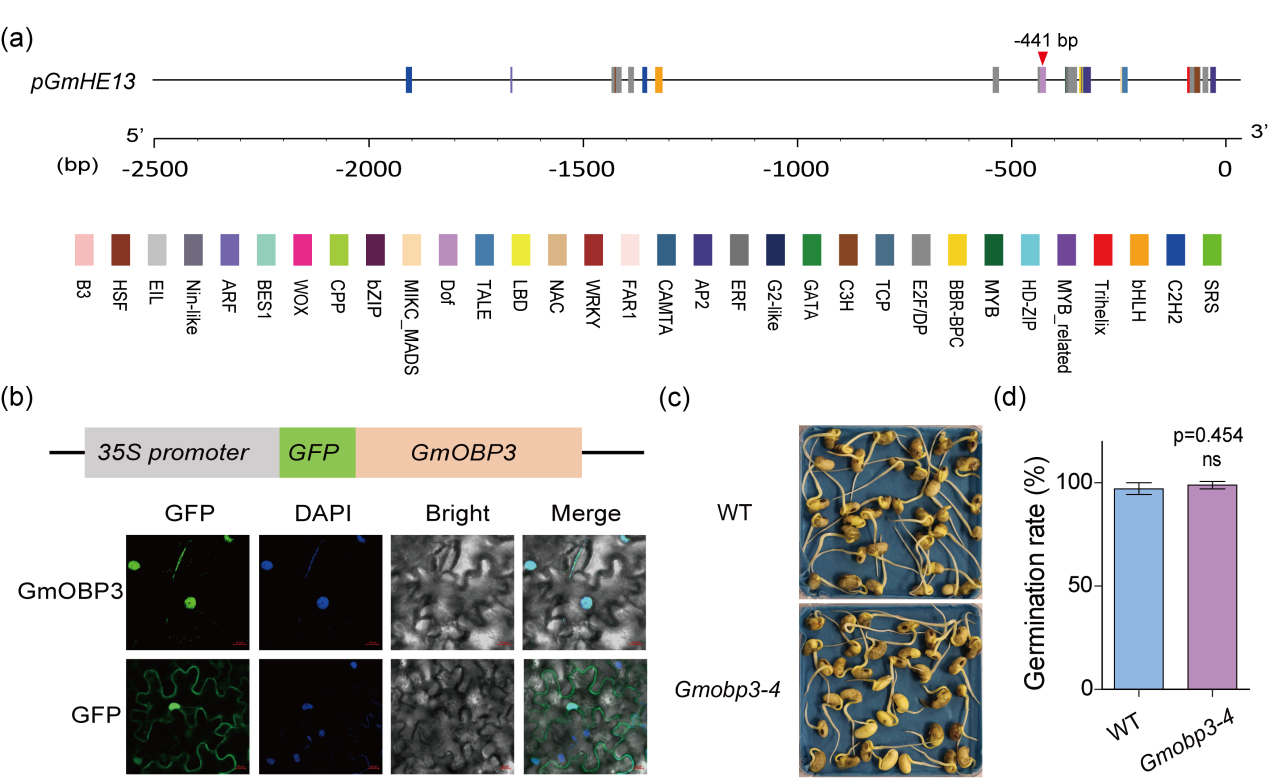


**Figure S13 Expression pattern and subcellular localization of GmOBP3.** **(a)** Prediction of cis-elements in the promoter of *GmHE13* using the online tool PlantTFDB (https://plantregmap.gao-lab.org/). Red triangle indicates a GmOBP3 binding site at -441 bp in the *GmHE13* promoter. **(b)** Subcellular localization of GFP-GmOBP3 in tobacco mesophyll cells. Scale bars, 20 μm. DAPI staining indicates the location of the nuclei. **(c)** Representative photos of WT and *Gmobp3-4* mutant seed germination under dark conditions. **(d)** Germination rates in (c), with n = 3 biological replicates, 36 seeds each. ns, not significant (*p* > 0.05, Student’s *t*-test).
